# Compost and Phosphorus/Potassium-Solubilizing Fungus Effectively Boosted Quinoa’s Physio-Biochemical Traits, Nutrient Acquisition, Soil Microbial Community, and Yield and Quality in Normal and Calcareous Soils

**DOI:** 10.3390/plants12173071

**Published:** 2023-08-27

**Authors:** Samah M. Youssef, Ahmed Shaaban, Abdelsattar Abdelkhalik, Ahmed R. Abd El Tawwab, Laila R. Abd Al Halim, Laila A. Rabee, Khairiah Mubarak Alwutayd, Reda M. M. Ahmed, Rahaf Alwutayd, Khaulood A. Hemida

**Affiliations:** 1 Horticulture Department, Faculty of Agriculture, Fayoum University, Fayoum 63514, Egypt; smy00@fayoum.edu.eg (S.M.Y.); aga04@fayoum.edu.eg (A.A.); 2 Agronomy Department, Faculty of Agriculture, Fayoum University, Fayoum 63514, Egypt; 3 Soil and Water Department, Faculty of Agriculture, Fayoum University, Fayoum 63514, Egypt; ara08@fayoum.edu.eg (A.R.A.E.T.); rmm11@fayoum.edu.eg (R.M.M.A.); 4 Agricultural Microbiology Department, Faculty of Agriculture, Fayoum University, Fayoum 63514, Egypt; lra00@fayoum.edu.eg; 5 Department of Food Science and Technology, Faculty of Agriculture, Fayoum University, Fayoum 63514, Egypt; lar00@fayoum.edu.eg; 6 Department of Biology, College of Science, Princess Nourah bint Abdulrahman University, Riyadh 11671, Saudi Arabia; kmalwateed@pnu.edu.sa; 7 Department of Information Technology, College of Computer and Information Science, Princess Nourah bint Abdulrahman University, Riyadh 11671, Saudi Arabia; 441004641@pnu.edu.sa; 8 Botany Department, Faculty of Science, Fayoum University, Fayoum 63514, Egypt; kah00@fayoum.edu.eg

**Keywords:** plant–microbe interactions, bioorganic mineral fertilization, high CaCO_3_-affected soil, quinoa’s nutritional quality, ionic homeostasis, antioxidant capacity

## Abstract

Calcareous soil had sufficient phosphorus and potassium (PK) in different forms due to the high contents of PK-bearing minerals; however, the available PK state was reduced due to its PK-fixation capacity. Compost, coupled with high PK solubilization capacity microbes, is a sustainable solution for bioorganic fertilization of plants grown in calcareous soil. A 2-year field experiment was conducted to investigate the effect of compost (20 t ha^−1^) with *Aspergillus niger* through soil drenching (C-AN) along with partial substitution of PK fertilization on quinoa performance in normal and calcareous soils. Treatments included PK_100%_ (72 kg P_2_O_5_ ha^−1^ + 60 kg K_2_O ha^−1^ as conventional rate), PK_100%_+C-AN, PK_75%_+C-AN, PK_50%_+C-AN, PK_25%_+C-AN, and only C-AN in normal and calcareous soils. Results showed that C-AN and reduced PK fertilization (up to 75 or 50%) increased photosynthetic pigments and promoted nutrient acquisition in quinoa grown in calcareous soil. Reduced PK fertilization to 75 or 50% plus C-AN in calcareous soil increased osmoprotectants, nonenzymatic antioxidants, and DPPH scavenging activity of quinoa’s leaves compared to the PK_0%_+C-AN treatment. The integrative application of high PK levels and C-AN enhanced the quinoa’s seed nutritional quality (i.e., lipids, carbohydrates, mineral contents, total phenolics, total flavonoids, half maximal inhibitory concentration, and antiradical power) in calcareous soil. At reduced PK fertilization (up to 75 or 50%), application of compost with *Aspergillus niger* through soil drenching increased plant dry weight by 38.7 or 53.2%, hectoliter weight by 3.0 or 2.4%, seed yield by 49.1 or 39.5%, and biological yield by 43.4 or 33.6%, respectively, compared to PK_0%_+C-AN in calcareous soil. The highest P-solubilizing microorganism’s population was found at PK_0%_+C-AN in calcareous soil, while the highest *Azotobacter* sp. population was observed under high PK levels + C-AN in normal soil. Our study recommends that compost with *Aspergillus niger* as a bioorganic fertilization treatment can partially substitute PK fertilization and boost quinoa’s tolerance to salt calcareous-affected soil.

## 1. Introduction

Areas with arid and semiarid climates often have soils that are considered calcareous, which pose a problem for crop production. These soils are estimated to account for more than a third of the Earth’s land surface [1]. Calcareous soil contains large quantity of calcium carbonate (CaCO_3_) exceeds 14–17% as total CaCO_3_ or 4–7% as active CaCO_3_ [2]. This substantial proportion of CaCO_3_, regardless of its form, alters soil’s different properties and hence limits its cropping range and productivity. However, crusting, the composition of a hard, cemented layer of CaCO_3_ particles on soil surface, is the major problem of calcareous soils. Soil crust affects soil properties by retarding seed germination and slowing down root development [3], resulting in poor infiltration and accelerating surface runoff [4,5]. Calcareous soil additionally has pH values of soil paste ranging from 7.4 to 9.2 [6,7], low organic matter content (<0.4%), and mostly decreases with depth [8]. High CaCO_3_ of calcareous soil fixes the nutrient availability (e.g., Zn^+2^, Fe^+2^, Mn^+2^, B, Mg^+2^, P, K^+^, and N) affecting plant performance [9]. Thus, applying an integrated management package is essential to enhance calcareous soil quality and achieve sustainable productivity [1].

Plants need phosphorus (P) as the second most essential macronutrient. However, many soils have low amounts of phosphate that plants can use, even if they have high amounts of total P [10,11]. Increased pH and CaCO_3_ levels lead to direct and/or indirect nutrient immobilization. Higher pH boosts the transformation of P into insoluble forms [12], consequently reducing P availability and P use efficiency [13], which strongly impacts soil productivity. Microorganisms that can make insoluble P soluble can act as biofertilizers to use the P stored in soils and increase the amount of soluble P [14]. Inoculation of fungi that can solubilize phosphate can improve plant growth and P uptake, as shown in pot experiments [15] and field trials [16]. Some *Aspergillus* species have already been described for their P solubilization ability and potential use as soil P solubilizers from different sources (i.e., rock phosphate, aluminum phosphate, tricalcium phosphate, etc.), thus supplying available P and enhancing plant growth [17,18]. Additionally, the United States Food and Drug Administration generally regards the 19 *Aspergillus* species in the section Nigri, which includes *A. niger* [19], as safe. 

Potassium (K) is the key essential macronutrient for biological growth and development [20]. However, plants take up K from the soil solution as K ions, and the quantities of soluble K in soil are usually very low, while most of the K in soil are rocks, minerals, and other deposits that are not soluble [21,22]. Despite these sources making up the largest pools of K in soil, under suitable conditions, they can be dissolved and become accessible for plants. Calcareous soils, however, have minor content (<150 mg kg^−1^ soil) of available K, reflecting their deficiency [21]. Microorganisms are vital in the natural K cycle [23,24]. Some fungal strains possess the capacity to dissolve rock potassium and potassium aluminum silicate [25]. Filamentous fungal species, especially those from *Aspergillus* and *Penicillium*, produce large amounts of organic acids and have a key role in the K cycle by dissolving insoluble substrates containing K [26,27]. These findings support the use of K-dissolving fungi to dissolve native K minerals in the soil in an environmentally friendly and more sustainable approach [28]. Furthermore, microorganisms including many K-dissolving fungi can enhance plant growth by secreting phytohormones such as indole-3-acetic acid (IAA) [29]. Therefore, the interaction between applied P or K fertilizers and soil minerals results in low nutrient use efficiency [30]. 

However, to meet plant nutrient requirements in calcareous soils, huge quantities of mineral fertilizers must be used, creating an economic and environmental risk [31]. However, to achieve sustainability of arable lands and to replenish the productivity of such degraded soils, it is critical to adopt ecofriendly and cost-effective solutions for the efficient exploitation of P and K fertilizers. *Aspergillus niger* is a multipurpose fungus involved in the solubilization of K and P [32] as well as the stimulation of phytohormone biosynthesis [33,34]. Application of *A. niger* exhibited improvements in soil quality, microbial community, and lettuce yield in barrier soil [35]. *A. niger* can dissolve many elements such as P, K^+^, Ca^2+^, and other elements present in rocks and minerals, releasing them in a form that is easily absorbed by plants. In addition, *A. niger* was found to be synthesized in plant growth-promoting and growth-protecting molecules such as sesquiterpenes (*β*-caryophyllene), IAA, and gibberellins, as well as 2-carboxymethyl-3-hexyl-maleic anhydride and 2-methylene-3-hexyl-butanedioic acid [34]. 

Adding compost with nutrient-solubilizing fungi have a positive effect on soil properties and the availability of nutrients (especially P and K) to plants under calcareous soil conditions. Compost application on calcareous soil has been described to improve soil physicochemical properties and intensifies microbial population and its activity [2,36]. It has also been reported that compost raises soil nutrient availability through decreasing soil pH, maintains soil health and fertility, and increases crop yields [37,38]. Recently, using microorganisms with compost as a bioorganic fertilizer outperformed conventional compost, which eventually boosted crop yield. Furthermore, microorganism-enriched compost has increased the soil nutrient availability compared with the unenriched compost [39]. When nutrient-solubilizing microorganisms are added or inoculated with compost, the enhanced bio-input obtained offers extra long-term benefits when it is applied to the soil [40]. Therefore, combining application of bioorganic fertilizer (herein; *A. niger* with compost) and reduced chemical fertilization promises to reduce the use of mineral K and P fertilizers in an ecofriendly and more sustainable manner.

Quinoa (*Chenopodium quinoa* Willd.) is a herbaceous, pseudo-cereal C3 tetraploid halophyte crop belonging to the *Chenopodiaceae* family [41,42]. Quinoa grain production has high economic value due to its composition of protein, oil, essential amino acids, carbohydrates, fatty acids, vitamins, and minerals. With protein ranges between 11 and 19%, it plays a vital role in boosting the immune system, thus fighting various diseases [43,44]. The whole quinoa plant can be used as livestock feed, and the leaves can be eaten as leafy vegetables as a source of nutrients. Despite quinoa’s ability to grow under harsh conditions [45,46], it is not well known whether or not quinoa can grow effectively in calcareous soil. 

Although many studies have been conducted on compost supplanted with nutrient-solubilizing microbes, scarce literature is found investigating the influence of compost with P- and/or K-solubilizing fungi (e.g., *A. niger*) with different PK levels on agronomic, physio-biochemical attributes, and seed quality of quinoa seeded in calcareous soil. This study hypothesized that inoculation of compost with nutrient-solubilizing fungus would improve quinoa growth and yield under calcareous soil conditions by boosting nutrient acquisition, osmoprotectants, and antioxidants. It is also expected that integrative application of PK-solubilizing *A. niger* could partially substitute PK chemical fertilizer. Therefore, the current study aimed at investigating the impact of co-application of compost with P- and K-solubilizing fungus under different mineral PK levels on photosynthetic pigments, osmolyte and antioxidant accumulation, nutrient acquisition, growth, seed yield, and the nutritional quality of quinoa under normal and calcareous soil conditions. 

## 2. Results

### 2.1. Photosynthetic Pigments

The illustrated data in Table S5 reveal that the leaf chlorophylls, carotenoids, and total photosynthetic pigments were similar during both growing seasons. Regarding the soil type effect, data exhibited that the plants grown in calcareous soil recorded lower contents of leaf chlorophyll *a*, chlorophyll *b*, total chlorophylls, carotenoids, and total photosynthetic pigments than those grown in normal soil. Regarding the co-applied PK+C-AN effect, there was an increase in the aforementioned photosynthetic parameters when increasing the level of PK plus C-AN, highlighting that the highest values were obtained under PK_100%_+C-AN, while the lowest values were obtained under PK_0%_+C-AN. In the case of soil type and PK+C-AN interaction (Table 1), application of PK_100%_+C-AN to quinoa plants grown in normal soil resulted in the highest values of chlorophyll *a*, total chlorophylls, carotenoids, and total photosynthetic pigments, whereas the application of PK_0%_+C-AN to plants grown in calcareous soil resulted in the lowest values. However, application of *A. niger* with compost and reduced PK fertilization (75 or 50% of PK) and reversed the negative impacts of calcareous soil on quinoa plants by increasing chlorophyll *a* by 24.8 or 17.4%, total chlorophylls by 25.1 or 17.1%, carotenoids by 26.5 or 14.5%, and total photosynthetic pigments by 25.4 or 16.7% compared to the plants treated with PK_0%_+C-AN. They also recorded similar or higher values to the quinoa plants treated only with full PK fertilization (PK_100%_) in normal soil (Table 1).

### 2.2. Total Soluble Proteins, Osmoregulatory Compounds, and Nonenzyme Antioxidants

**Figure 1:**
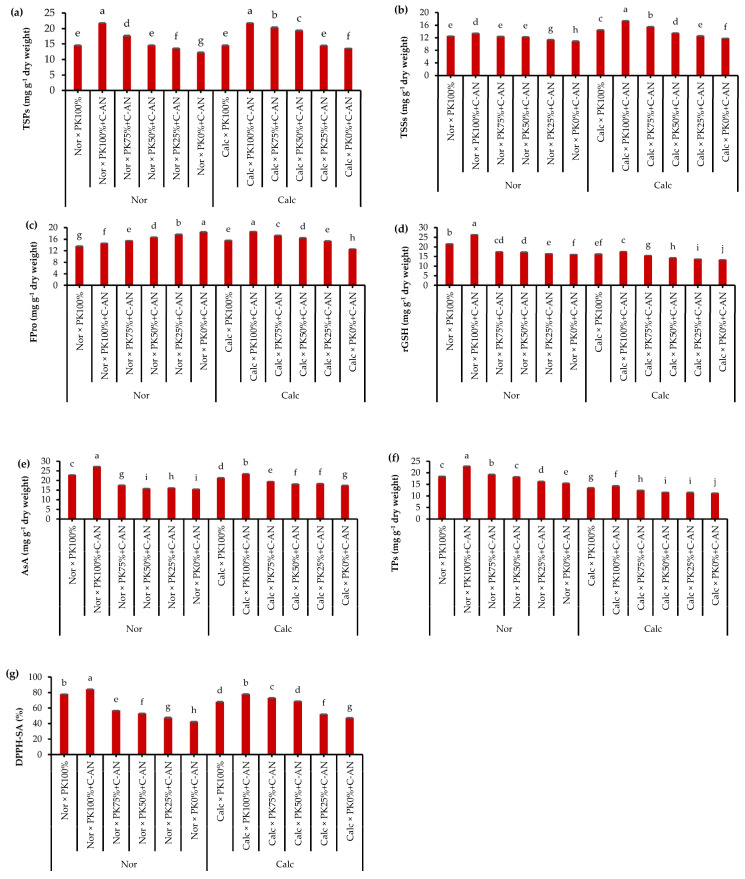
Interactive effect of soil type (ST) and compost with phosphate (P)–potassium (K)-solubilizing *Aspergillus niger* (PK+C-AN) level on leaf biochemical attributes, e.g., (**a**) total soluble proteins (TSPs), (**b**) total soluble sugars (TSSs), (**c**) free proline (FPro), (**d**) reduced glutathione (rGSH), (**e**) ascorbic acid (AsA), (**f**) total phenolics (TPs), and (**g**) 2,2-diphenyl-1-picrylhydrazyl-scavenging activity (DPPH-SA). PK_100%_ = 72 kg P_2_O_5_ ha^−1^ + 60 kg K_2_O ha^−1^, PK_75%_ = 54 kg P_2_O_5_ ha^−1^ + 45 kg K_2_O ha^−1^, PK_50%_ = 36 kg P_2_O_5_ ha^−1^ + 30 kg K_2_O ha^−1^, PK_25%_ = 18 kg P_2_O_5_ ha^−1^ + 15 kg K_2_O ha^−1^, PK_0%_ = 0 kg P_2_O_5_ ha^−1^ + 0 kg K_2_O ha^−1^, and compost was added with a rate of 20 t ha^−1^. Normal (Nor) and calcareous (Calc). Each bar is expressed as the mean ± standard error of the mean (*n* = 3). Bars labeled with same letters are not significantly different according to the Duncan test (*p* ≤ 0.05). Values based on average of 2021/22 and 2022/23 seasons.

In both seasons, the total soluble proteins, total soluble sugars, proline, reduced glutathione (rGSH), ascorbic acid (AsA), total phenolics, and total antioxidant activity in quinoa leaves were at similar levels (Table S6). As for soil type, quinoa plants grown under calcareous soil had higher concentrations of total soluble proteins, total soluble sugars, AsA, and total antioxidant activity compared to those grown under normal soil that had higher rGSH and total phenolics contents. With respect to the PK+C-AN effect, it was observed that there is a general trend of increasing the contents of total soluble proteins, total soluble sugars, proline, rGSH, AsA, total phenolics, and total antioxidant activity in line with increasing the PK level when fortified with the C-AN (Figure 1). In this regard, the highest and the lowest values of the abovementioned biochemical attributes corresponded with the PK_100%_+C-AN and PK_0%_+C-AN treatments, respectively. Application of PK plus C-AN generated a stimulatory action on the quinoa plant cultivated in calcareous soil. Contextually, highlighting that PK_75%_+C-AN- or PK_50%_+C-AN-treated calcareous soil showed higher total soluble proteins (50.0 or 42.6%), total soluble sugars (31.4 or 14.4%), proline (37.3 or 31.0%), rGSH (16.5 or 7.5%), AsA (11.4 or 4.0%), total phenolics (10.0 or 36%), and total antioxidant activity (54.2 or 44.7%), respectively, compared to the PK_0%_+C-AN treatment. Additionally, at reduced PK fertilization (75 or 50%), compost with *A. niger* increased osmoprotectants and antioxidants more than those recorded under full PK fertilization without C-AN application in calcareous soil (Figure 1).

### 2.3. Quinoa Leaf Nutrient Contents

**Figure 2:**
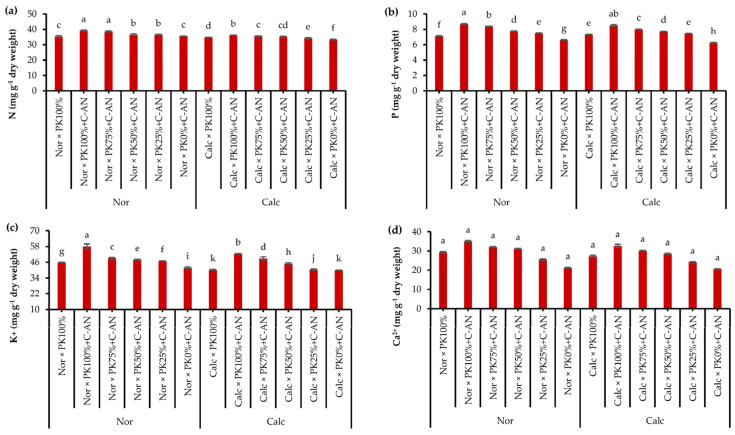

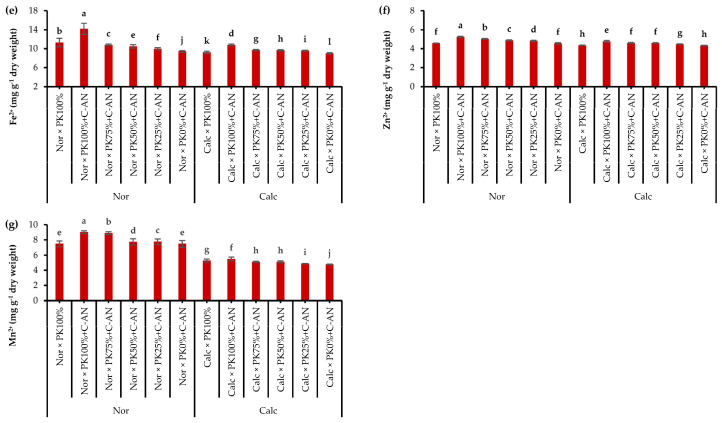
Interactive effect of soil type (ST) and compost with phosphate (P)–potassium (K)-solubilizing Aspergillus niger (PK+C-AN) level on leaf nutrients, e.g., nitrogen—N (**a**), phosphorus—P (**b**), potassium—K^+^ (**c**), calcium—Ca^2+^ (**d**), iron—Fe^2+^ (**e**), zinc—Zn^2+^ (f), and manganese—Mn^2+^ (g) contents. PK_100%_ = 72 kg P_2_O_5_ ha^−1^ + 60 kg K_2_O ha^−1^, PK_75%_ = 54 kg P_2_O_5_ ha^−1^ + 45 kg K_2_O ha^−1^, PK_50%_ = 36 kg P_2_O_5_ ha^−1^ + 30 kg K_2_O ha^−1^, PK_25%_ = 18 kg P_2_O_5_ ha^−1^ + 15 kg K_2_O ha^−1^, PK_0%_ = 0 kg P_2_O_5_ ha^−1^ + 0 kg K_2_O ha^−1^, and compost was added with a rate of 20 t ha^−1^. Normal (Nor) and calcareous (Calc). Each bar is expressed as the mean ± standard error of the mean (*n* = 3). Bars labeled with same letters are not significantly different according to the Duncan test (*p* ≤ 0.05). Values based on average of 2021/22 and 2022/23 seasons.

Data of quinoa leaf macro- and micronutrient analysis in response to growing season, soil type, PK+C-AN application, and soil type and PK+C-AN interaction are illustrated in Table S7. The N, K^+^, and Fe^2+^ acquisitions in quinoa were higher in the S_I_ season, while the Mn^2+^ was higher in the quinoa plant grown during SII. The contents of N, P, K^+^, Ca^2+^, Fe^2+^, Zn^2+^, and Mn^2+^ were higher in quinoa leaves grown in normal soil than in calcareous soil. Regarding the PK+C-AN level, a significant increase in macro- and micronutrient acquisition was observed, corresponding to an increase in the PK level plus C-AN. As for soil type and PK+C-AN level interaction, adding PK_100%_+C-AN under normal soil resulted in the maximum nutrient acquisition, while adding PK_0%_+C-AN under calcareous soil achieved the lowest values of nutrient acquisition. Calcareous soil treated with PK_75%_+C-AN or PK_50%_+C-AN increased the contents of N by 6.6 or 5.4%, P by 28.5 or 23.7%, K^+^ by 22.0 or 12.5%, Ca^2+^ by 46.6 or 38.3%, Fe^2+^ by 7.8 or 7.1%, Zn^2+^ by 6.5 or 6.2%, and Mn^2+^ by 7.1 or 7.2% compared to the PK_0%_+C-AN treatment, and also recorded higher values than the PK_100%_ treatment (Figure 2).

### 2.4. Seed Quality

In the case of soil type effect, seeds of quinoa plant grown in normal soil had higher protein, fiber, total phenolic compounds, total flavonoid compounds, and half maximal inhibitory concentration whereas those grown in calcareous soil recorded higher ash, lipid, water-soluble carbohydrates, and antiradical power (Tables S8 and S9). As for PK+C-AN level, the highest seed protein, ash, fiber, and water-soluble carbohydrates corresponded to the PK_25%_+C-AN, PK_100%_+C-AN, PK_0%_+C-AN, and PK_100%_ treatment, respectively, while the seed lipid content was not varied by the PK+C-AN level. Additionally, the total phenolic compounds, total flavonoid compounds, and half maximal inhibitory concentration were significantly increased with increasing PK+C-AN level.

As for soil type and PK+C-AN level interaction, the seeds of quinoa plant grown in normal soil and treated with PK_100%_+C-AN had the highest protein content, while the highest ash contents were observed from the seeds obtained from the plant treated with PK_100%_+C-AN under calcareous soil. The maximum seed lipid and fiber content corresponded to the plant treated with PK_25%_+C-AN and PK_0%_+C-AN under normal soil as well as the greatest amount of water-soluble carbohydrates (Table 2). The highest values of total phenolic compounds, total flavonoid compounds, and half maximal inhibitory concentration were obtained under normal soil × PK_100%_+C-AN treatment while the highest value of antiradical power was obtained under calcareous soil × PK_100%_+C-AN treatment (Table 3). In comparison to the PK_0%_+C-AN treatment, quinoa plants grown under calcareous soil treated with PK_75%_+C-AN or PK_50%_+C-AN generated positive results such as increased total phenolic compounds, total flavonoid compounds, half maximal inhibitory concentration, and antiradical power by 32.6 or 34.4%, 64.4 or 37.0%, 40.6 or 34.4%, and 36.1 or 27.3%, respectively (Table 3).

Calcareous soil had a negative impact on the mineral contents of quinoa seeds, given that the P, K+, Ca^2+^, Mg^2+^, Na^+^, Fe^2+^, and Zn^2+^ levels significantly decreased compared to quinoa grown in normal soil. Regardless of soil type, increasing the level of PK+C-AN significantly increased seed’s mineral content (Table S10). Under calcareous soil, application of high PK+C-AN levels mediated ameliorating impact on quinoa plant (Table 4). Additionally, the PK_75%_+C-AN or PK_50%_+C-AN treatments, under calcareous soil conditions, significantly (except for Ca^2+^) increased the aforementioned seed mineral content by 82.6 or 73.9%, 22.3 or 12.3%, 50.5 or 47.6%, 21.2 or 13.6%, 62.5 or 50.0%, and 19.5 or 17.1%, respectively, compared to the PK_0%_+C-AN treatment (Table 4).

### 2.5. Microbial Community

**Figure 3:**
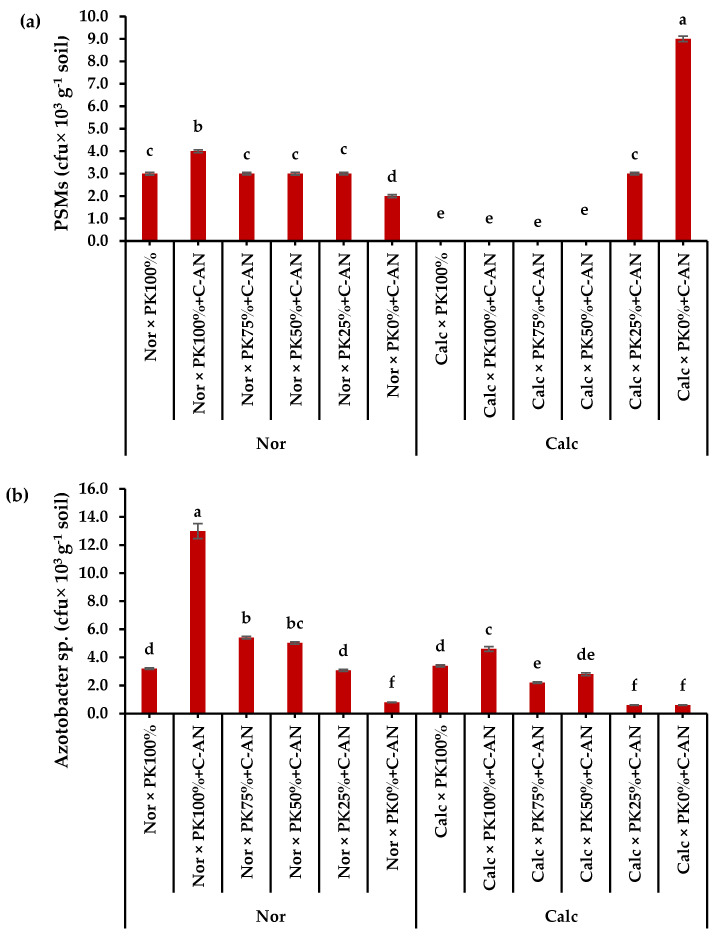
Interactive effect of soil type (ST) and compost with phosphate (P)–potassium (K)-solubilizing *Aspergillus niger* (PK+C-AN) level on total microbial community, e.g., (**a**) phosphorus-solubilizing microorganisms (PSMs) and (**b**) *Azotobacter* sp. at the end of experiment in rhizosphere soil of quinoa grown in (SI) 2021/22 and (SII) 2022/23 winter seasons. PK_100%_ = 72 kg P_2_O_5_ ha^−1^ + 60 kg K_2_O ha^−1^, PK_75%_ = 54 kg P_2_O_5_ ha^−1^ + 45 kg K_2_O ha^−1^, PK_50%_ = 36 kg P_2_O_5_ ha^−1^ + 30 kg K_2_O ha^−1^, PK_25%_ = 18 kg P_2_O_5_ ha^−1^ + 15 kg K_2_O ha^−1^, PK_0%_ = 0 kg P_2_O_5_ ha^−1^ + 0 kg K_2_O ha^−1^, and compost was added with a rate of 20 t ha^−1^. Normal (Nor) and calcareous (Calc). Each bar is expressed as the mean ± standard error of the mean (*n* = 3). Bars labeled with same letters are not significantly different according to the Duncan test (*p* ≤ 0.05). Values based on average of 2021/22 and 2022/23 seasons.

The total population of phosphate-solubilizing microorganisms and *Azotobacter* sp. in the rhizosphere soil of quinoa were higher in normal soil than in calcareous soil (Table S11). The highest phosphate-solubilizing microorganisms corresponded to the PK_0%_+C-AN level, while the highest *Azotobacter* sp. quantity was observed in PK_100%_+C-AN (Table S11). A significant interaction was observed when adding PK+C-AN in both soil types. The highest phosphate-solubilizing microorganisms were found in calcareous soil at PK_0%_+C-AN levels, whereas higher PK+C-AN levels did not produce any activity, compared to when applied in normal soil. The highest *Azotobacter* sp. population was observed under PK_100%_+C-AN followed by PK_75%_+C-AN then PK_50%_+C-AN in normal soil (Figure 3).

### 2.6. Quinoa’s Growth, Yield and Yield-Related Attributes, and Harvest Index

Calcareous soil significantly decreased quinoa plant height, hectoliter weight, seed yield, and biological yield, but resulted in higher HI in comparison to plant grown under normal plant (Table S11). Statistical analysis showed significant increase in the plant height, hectoliter weight, seed yield, and biological yield when increasing the PK+C-AN level. The HI was lower in the PK_25%_+C-AN treatment, while the other levels recorded similar results (Table S11). There were significant differences in the plant height, plant dry weight, hectoliter weight, seed yield, and biological yield between PK+C-AN levels under calcareous and normal soil (Table 5). The application of PK_100%_+C-AN in normal soil and PK_0%_+C-AN in calcareous soil yielded the highest and lowest values of all growth and yield parameters, respectively. At reduced PK fertilization, co-application of *A. niger* and compost in calcareous soil generated positive results, with similar or higher recorded values to the PK_100%_ in normal soil. Also, they increased plant height by 11.7 or 10.5%, hectoliter weight by 3.0 or 2.4%, seed yield by 49.1 or 39.5%, and biological yield by 43.4 or 33.6%, respectively, compared to the application of PK_0%_+C-AN in calcareous soil (Table 5).

### 2.7. Relationship among Applied Treatments and Studied Attributes

**Figure 4:**
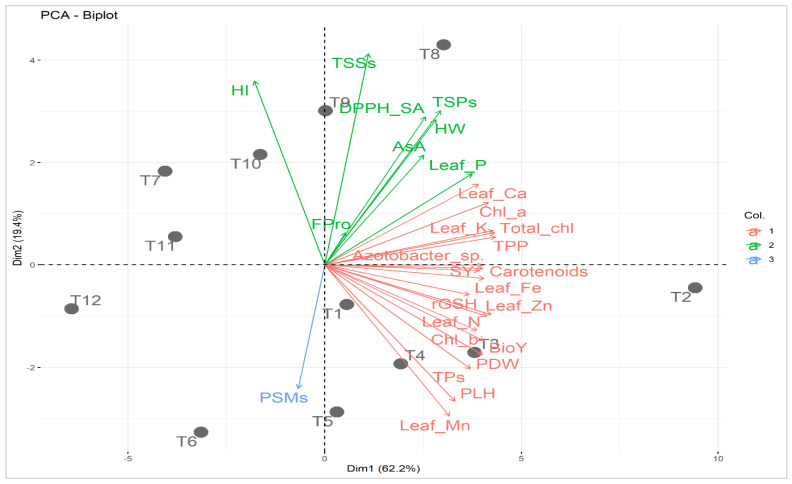
Principal component biplot (PCA) for applied treatments (soil type + bio-organo-mineral applications) and studied attributes. T1: Nor + PK_100%_ = the normal soil received the full recommended dose (FRD) of phosphorus (P) and potassium (K) fertilizers; T2: Nor + PK_100%_+C-AN = the normal soil received PK_100%_ + 20 t compost ha^−1^ + PK-solubilizing *Aspergillus niger*; T3: Nor + PK_75%_+C-AN = the normal soil received 75% out of FRD of P and K fertilizers + 20 t compost ha^−1^ + PK-solubilizing *Aspergillus niger*; T4: Nor + PK_50%_+C-AN = the normal soil received 50% out of FRD of P and K fertilizers + 20 t compost ha^−1^ + PK-solubilizing *Aspergillus niger*; T5: Nor + PK_25%_+C-AN = the normal soil received 25% out of FRD of P and K fertilizers + 20 t compost ha^−1^ + PK-solubilizing *Aspergillus niger*; T6: Nor + PK_0%_+C-AN = neither P nor mineral K fertilizer was added to the tested normal soil; T7: Calc + PK_100%_ = the calcareous soil received FRD of P and K fertilizers; T8: Calc + PK_100%_+C-AN = the calcareous soil received PK_100%_ + 20 t compost ha^−1^ + PK-solubilizing *Aspergillus niger*; T9: Calc + PK_75%_+C-AN = the calcareous soil received 75% out of FRD of P and K fertilizers + 20 t compost ha^−1^ + PK-solubilizing *Aspergillus niger*; T10: Calc + PK_50%_+C-AN = the calcareous soil received 50% out of FRD of P and K fertilizers + 20 t compost ha^−1^ + PK-solubilizing *Aspergillus niger*; T11: Calc + PK_25%_+C-AN = the calcareous soil received 25% out of FRD of P and K fertilizers + 20 t compost ha^−1^ + PK-solubilizing *Aspergillus niger*; T12: Calc + PK_0%_+C-AN = neither P nor mineral K fertilizer was added to the tested calcareous soil. Chl *a*: chlorophyll *a*; Chl *b*: chlorophyll *b*; Total chl: total chlorophyll; TPP: total photosynthetic pigments; TSPs: total soluble proteins; TSSs: total soluble sugars; Fpro: free proline; rGSH: reduced glutathione; AsA: ascorbic acid; TPs: total phenolics; DPPH-SA: 2,2-diphenyl-1-picrylhydrazyl-scavenging activity; N: nitrogen; P: phosphorus; K^+^: potassium; Ca^2+^: calcium; Fe^2+^: iron; Zn^2+^: zinc; Mn^2+^: manganese; PSMs: phosphorus-solubilizing microorganisms; PLH: plant height; PDW: plant dry weight; HW: hectoliter weight; SY: seed yield; BioY: biological yield; and HI: harvest index. Col.: color. Values based on average of 2021/22 and 2022/23 seasons. Each dot (•) indicates a treatment number.

Principal component analysis was carried out to study the relationship between applied treatments and studied attributes (Figure 4). The first two principal components described 81.6% of the variability. The PC1 displayed 62.2% of the variation and was correlated to soil type (i.e., normal and calcareous; Figure 4). The PC1 divided the applied treatments based on soil type into two groups; treatments in the normal soil were located on the positive side while those of Calc soil were situated on the negative side. On the other hand, PC2 exhibited 19.4% of the variation and was related to mineral PK fertilization combined with C-AN at both soil types. Treatments of mineral PK fertilization combined with C-AN in normal soil were located at the bottom part of PC2 while those of Calc soil were located at the top. The increase in mineral PK fertilization from 0 to 100% corresponded with PC2 from the bottom to the top at both sections of soil type. The best treatments were assigned for T8 (PK_100%_+C-AN) followed by T9 (PK_75%_+C-AN) in calcareous soil and T2 (Nor+PK_100%_+C-AN) followed by T3 (PK_75%_+C-AN) in normal soil. The adjacent vectors of studied characters reflected a positive correlation with each other. Quinoa seed yield and its related-attributes (e.g., PLH, PDW, HW, and BioY) displayed a strong relationship with photosynthetic pigments (e.g., chl *a*, chl *b*, total chl, carotenoids, TPP), leaf nutrients (e.g., N, P, K^+^, Ca^2+^, Fe^2+^, Zn^2+^, and Mn^2+^), and bacterial (e.g., PSMs and *Azotobacter* sp.) count in soil. Otherwise, seed yield and its related attributes exhibited a negative correlation with osmoprotectants and nonenzymatic antioxidants (e.g., TSPs, TSSs, FPro, rGSH, AsA, and TPs), as well as DPPH-SA.

**Figure 5:**
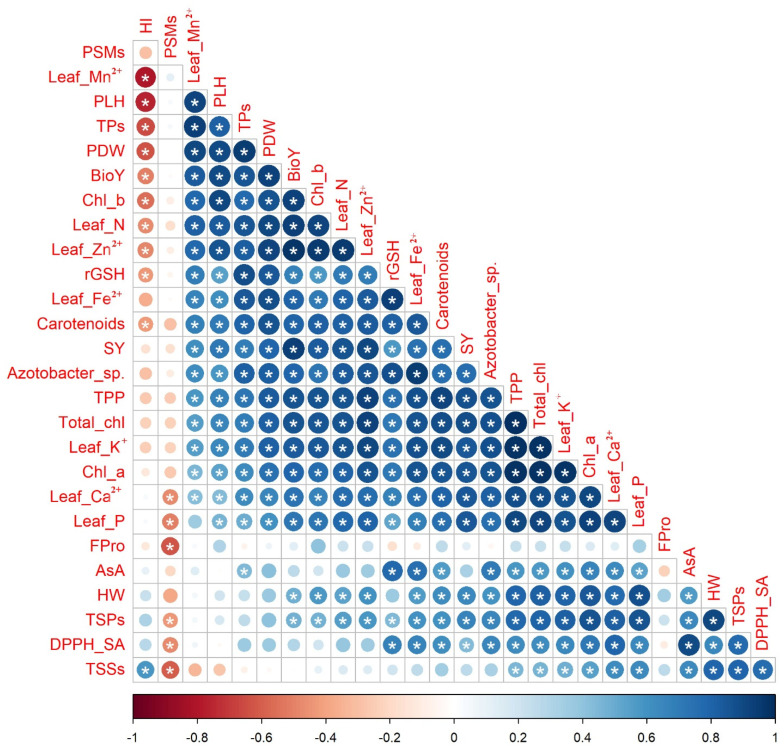
Pairwise comparisons among selected quinoa crop traits based on the data of soil types × bio-organo-mineral treatments shown in the correlogram are based on Pearson’s correlation coefficients. The circle color in the correlogram corresponds to the correlation coefficient, wherein a positive correlation coefficient is closer to 1 (purple end of the scale) and a negative correlation coefficient is closer to −1 (red end of the scale). The circle size matches the significance level. Chl *a*: chlorophyll *a*; Chl *b*: chlorophyll *b*; Total chl: total chlorophyll; TPP: total photosynthetic pigments; TSPs: total soluble proteins; TSSs: total soluble sugars; Fpro: free proline; rGSH: reduced glutathione; AsA: ascorbic acid; TPs: total phenolics; DPPH-SA: 2,2-diphenyl-1-picrylhydrazyl-scavenging activity; N: nitrogen; P: phosphorus; K^+^: potassium; Ca^2+^: calcium; Fe^2+^: iron; Zn^2+^: zinc; Mn^2+^: manganese; PSMs: phosphorus-solubilizing microorganisms; PLH: plant height; PDW: plant dry weight; HW: hectoliter weight; SY: seed yield; BioY: biological yield; and HI: harvest index. (*) refers to significant Person’s correlation at *p* ≤ 0.05. Values based on average of 2021/22 and 2022/23 seasons.

The Pearson correlation coefficients, which are displayed in the correlogram found in Figure 5, were calculated to find out the level of association among selected attributes of quinoa plants based on the data obtained from the interaction of soil type and bio-organo-mineral treatments as averaged over both seasons. Significant, whether positive or negative with a *p*-value ≤ 0.05, or nonsignificant correlations were noticed between SY and each of the other chosen quinoa crop attributes. The SY had significant (*p* ≤ 0.05) positive correlations with each of the photosynthetic pigments (e.g., Chl *a*, Chl *b*, total chl, carotenoids, and TPP), osmoprotectants and nonenzymatic antioxidants (e.g., TSPs, rGSH, and TPs), leaf nutrients (e.g., N, P, K^+^, Ca^2+^, Fe^2+^, Zn^2+^, and Mn^2+^), soil *Azotobacter* sp. population count, and seed yield-related attributes (e.g., PLH, PDW, HW, and BioY), while being nonsignificantly negatively correlated with HI.

## 3. Discussion

Calcareous soils are commonly found in arid areas with hot weather, drought, and low precipitation [2,47]. This soil is characterized by poor hydro- and physicochemical properties [48,49], as well as low P and K availability [2]; therefore, microorganisms, particularly bacteria and fungi, help to chelate, acidify, and process P and K into soluble forms [50,51,52]. Hence, the application of compost supplemented with *A. niger* as a bio-organic fertilizer and reduced chemical fertilizers could be an important approach for sustainable agriculture development [53].

In the present study, quinoa plants were cultivated in calcareous soil, leading to physio-biochemical abnormalities. Quinoa plants responded to the stress induced by calcareous soil by reducing their photosynthesis pigments and biochemical traits (Tables S5 and S6), nutrient acquisition (Table S7), and, consequently, dry biomass and seed yield (Table S11). Similar observations have also been reported in sunflower plants grown in calcareous soil [54]. Moreover, the incorporation of bioorganic fertilizer into soil has been widely used to improve its hydrological, physicochemical, and biota properties, as well as nutrients’ availability and crop productivity [55,56]. In this experiment, *A. niger* was inoculated with compost, which has the ability to secrete tartaric acid, oxalic acid, and citric acid as well as dissolve elements in rocks and minerals, releasing the elements in a form that plants can easily absorb [57].

Our results revealed that the co-application of compost with *A. niger* as a bioorganic fertilizer product proportionally saved mineral P and K fertilizers and mitigated the harmful effect of calcareous soil on quinoa plants. In this context, combined *A. niger* with compost and reduced chemical fertilizer (75 or 50% of PK) achieved yields equivalent or superior to those obtained using full PK fertilizers without bioorganic fertilizers. Our findings were confirmed by those reported by Qaswar et al. [58], who found that application of organic amendments (e.g., wheat straw or swine manure) partially substituted NPK chemical fertilizers and improved soil quality and rice grain yield.

Under calcareous soil, increasing the PK rate plus C-AN enhanced the photosynthetic pigments. Although, the integrative application of *A. niger* and compost with reduced PK fertilization up to 25 or 50% (PK_75%_+C-AN or PK_50%_+C-AN) promoted the photosynthetic pigment contents compared to the application of sole full PK synthesized form. Bioorganic compost was found to improve root growth via secretion of auxins by microorganisms [31], along with the compost’s promotion of water holding capacity [48], which increases water absorption for higher chlorophyll biosynthesis [59]. Furthermore, increased chlorophyll and carotenoid content may be related to increased nutrient acquisition (Figure 2 and Table S7) in response to the co-application of compost with *A. niger* and higher PK fertilization.

It is clear that compost with microorganisms (in this case *A. niger*) can increase release elements in the soil and promote nutrient availability for plants in calcareous soil [60]. This was more evident in calcareous soil-treated C-AN, which increased the amount of osmoprotectants (Fpro, TSSs, and TSPs), nonenzymatic antioxidants (rGSH and AsA), and DPPH-scavenging activity (Figure 1 and Table S6). In this study, reduced PK fertilizers (75 or 50%) coupled with C-AN enhanced the accumulation of TSPs, FPro, and TSSs compared to full PK fertilization (PK_100%_) without C-AN in calcareous soil (Figure 1). These osmolytes are involved in maintaining cell turgor for osmotic adjustment, facilitating water and nutrient uptake, and thus stimulating the biosynthesis of photosynthetic pigments [61,62,63,64]. Moreover, accumulation of such compatible solutes together with nonenzymatic antioxidants (e.g., AsA, rGSH, and total phenolics; Figure 1) aids in stabilizing proteins, cellular membrane structure, and mitigating oxidative damage under stress conditions [49,65,66], consequently reflected in the increased yield and quality of quinoa. The AsA, rGSH, TPs, proline, and carotenoids are effective ROS scavengers that can protect stressed quinoa plant tissues under abiotic stress [67,68], and in this experiment, all increased in line with increasing PK levels when integrated with compost with *A. niger* (Tables S5 and S6). Furthermore, quinoa plants cultivated in calcareous soil treated with PK (particularly at higher levels) and C-AN displayed greater total antioxidant activity (68.6-77.9%) compared to those fertilized only with PK_100%_. The total antioxidant activity was analyzed as DPPH radical scavenging, which is broadly applied to screen antioxidant activity for the inhibition of lipid peroxidation [69].

Integrative compost with *A. niger* and higher PK fertilization enhanced the macro- and micronutrient acquisition in quinoa plant leaves under calcareous soil (Figure 2). In this respect, increasing nutrient acquisition may be linked to the impact of application of bioorganic fertilizers on soil pH, soil biota, and organic matter, boosting the breakdown and availability of macro- and micronutrients for plant uptake [70,71]. Integrative application of phosphate-solubilizing microorganisms with poultry manure was found to raise nutrient availability (i.e., Ca and P) in the calcareous soil [72]. After the addition of *A. niger* fermentation broth, the soil’s pH value decreased, and the soil’s effective P, exchangeable Ca^2+^, Mg^2+^, and effective Fe^2+^, Cu^2+^, and Zn^2+^ were efficiently liberated in the low pH condition, leading to an increase in the amount of nutrient elements present in the soil [58]. Partial exchange of mineral fertilization with organic fertilizers provides more macro- and micronutrient availability, resulting in stimulated photosynthetic capacity, growth, and yield of the common bean plant [73].

The improvements in nutrient acquisition by the integrative high PK level and C-AN were simultaneously associated with higher nutritive contents of quinoa seeds under calcareous soil, given the increased crude protein, crude lipid, carbohydrate (Table 2), and mineral contents (Table 4) of quinoa seeds. Moreover, the combination of *A. niger* with compost and reduced PK fertilization up to 75% enhanced the total phenolic compounds, total flavonoid compounds, half maximal inhibitory concentration, and antiradical power of quinoa seeds produced in calcareous soil (Table 3). Half maximal inhibitory concentration denotes the amount of a particular inhibitor that is needed to reduce a certain biological activity by 50%; thus, the higher the half maximal inhibitory concentration value, the less potent the inhibitor [74]. Additionally, the antiradical potential provides insights into lipid peroxidation and oxidative processes [75]. Therefore, our results indicated that the co-application of compost with *A. niger* plus reduced PK fertilization could enhance the chemical composition and antioxidant capacity of quinoa seeds grown in calcareous soil. Our findings are in harmony with the results reported by Youssef and Farag [76], and Yang et al. [77].

In the current research, the phosphate-solubilizing microorganism population increased in calcareous soil treated with C-AN without PK fertilization, while the *Azotobacter* sp. population increased in normal soil treated with high levels of PK fertilization plus C-AN (Figure 3a and b). Long-term NPK mineral fertilization decreased the community of phosphate-solubilizing microorganisms compared to long-term manure fertilization, which increased total carbon and N, regulated the soil pH, and provided favorable conditions for microorganisms [48,78].

In the present study, at reduced PK fertilization levels (50 or 75%), compost with inoculated *A. niger* notably increased the dry biomass accumulation, seed yield, and biological yield of quinoa plants compared to those only fertilized with mineral PK in calcareous soil (Table 5). These outcomes might be explained by an increase in osmolytes and antioxidant accumulation (Figure 1), nutrient acquisition (Figure 2), leading to an increase in photosynthetic pigments. In this context, our results agree with those reported by Qaswar et al. [58] on rice and Mohamed et al. [73] on common bean. Coupling poultry manure or farmyard manure with phosphate-solubilizing microbes elevated the maize shoot and root biomass over sole mineral source [79].

The current study demonstrated the efficacy of bioorganic fertilizers (in this case, *A. niger* with compost) as a valuable strategy for enhancing the performance and nutritive content of quinoa plants grown in calcareous soil. Therefore, in the future, growers can reduce P and K chemical fertilization up to 25–50% along with the application of bioorganic fertilizer for maintaining or even increasing quinoa yield in calcareous soil.

## 4. Materials and Methods

### 4.1. Experimental Location and Climatic Conditions

The current study was carried out at the Agriculture Faculty’s Research Farm, Fayoum province, Egypt (29°02′ and 29°35′ N latitudes and 30°23′ and 31°05′ E longitudes) during the 2021/22 and 2022/23 cropping seasons. Based on the aridity index [80], the region has a semiarid Mediterranean climate characterized by wet winters and arid summers. The main agrometeorological data recorded during the quinoa growing seasons (November–May) in 2021/22 and 2022/23 are presented in Figure S1. Based on their basic properties for 0–0.3 m depth (Table 6), determined according to the methodology described by Klute and Dirksen [81] and Page et al. [82], two different soils at the experimental sites were selected to conduct this experiment. The first is normal (i.e., noncalcareous) sandy loam and the other is calcareous sandy loam (˃15% CaCO_3_ content), which are referred to hereafter as normal and calcareous, respectively.

### 4.2. Fungi Isolation and Assessment of Its Phosphate and Potassium Solubilizing Ability

Fifteen fungal isolates were obtained from soil samples randomly collected from normal and calcareous agricultural soils in Fayoum Governorate, Egypt using potato dextrose agar (PDA) media. Morphologically distinct fungal colonies on the plates were chosen, purified through repeated culturing, relocated to a new PDA slant, and incubated at 4 °C.

Screening for phosphate and potassium solubilizing activity of all fungal isolates was carried out by allowing the fungi to grow on fresh Pikovskaya and Aleksandrov selective agar media, respectively, for 7 to 10 days at a temperature of 25 °C. The formation of a transparent halo zone surrounding the fungal colonies is indicated their ability to solubilize tri-calcium phosphate and potassium aluminum silicate, respectively [83,84]. Out of the 15 fungal isolates, the most promising phosphate and potassium solubilizing fungal isolate was selected based on its higher ability to solubilize phosphate and potassium, expressed as the solubilization index. The phosphate solubilization index (P-SI) was determined using the method of Premono et al. [85] and Elias et al. [86] as follows: P-SI = (colony + halo zone diameter)/colony diameter, while the potassium solubilization index was calculated via Khandeparkar’s selection ratio (KSR) according to Prajapati and Modi [87] as follows: KSR = diameter of clearance zone/diameter of fungus colony growth.

### 4.3. Molecular Identification of the Tested Fungal Isolate

**Figure 6:**
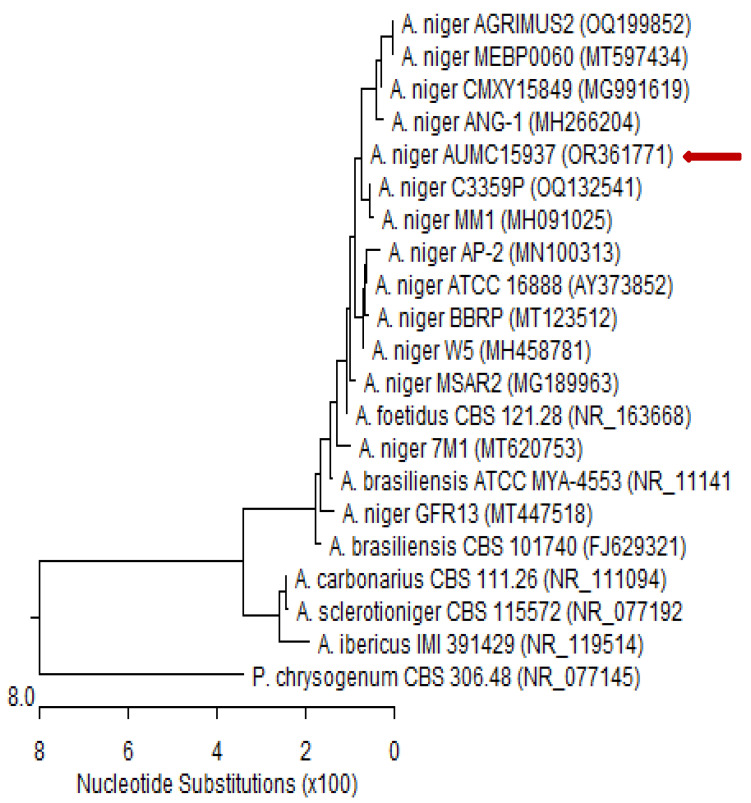
Phylogenetic tree of ITS sequences of rDNA from fungal isolate in the present study aligned with related sequences accessed from the Gen Bank. Phylogenetic tree based on ITS sequences of rDNA of *Aspergillus niger* AUMC15937 (red arrow) aligned with closely related sequences accessed from the GenBank. This strain showed 99.83–100% identity and 99–100% coverage with strain sequences of the same species. *Penicillium chrysogenum* is included in the tree as an outgroup strain. *Aspergillus niger* AUMC15937 registered in NCBI GenBank under accession no. OR361771. A. = *Aspergillus* and P. = *Penicillium*.

The fungal isolate was cultured in sterile Petri plates containing 20 mL of autoclaved Czapek’s yeast extract agar (CYA) and incubated at 28 °C for 5 days [88]. Patho-gene-spin DNA/RNA extraction kit (iNtRON Biotechnology Company, Sungnam, Korea) was used to extract DNA at the Molecular Biology Research Unit, Assiut University, Egypt. The extracted DNA was kept in a 1.5 mL autoclaved Eppendorf tube prior to shipping to SolGent Company, Daejeon, Republic of Korea, for polymerase chain reaction (PCR) and rRNA gene sequencing. The PCR was performed using forward 5′-TCCGTAGGTGAACCTGCGG-3′ (ITS1) and reverse 5′-TCCTCCGCTTATTGATATGC-3′ (ITS4) primers, which were incorporated into the reaction mixture. The purified PCR product was sequenced with the same primers with the addition of ddNTPs in the reaction mixture [89,90]. The Basic Local Alignment Search Tool (BLAST) available on the National Centre for Biotechnology Information (NCBI) database was used to analyze the acquired sequences. The phylogenetic sequence tree (Figure 6) was constructed using MegAlign software version 5.05 (DNA Star, Madison, WI, USA) according to Tamura et al. [91]. Based on ITS sequences of rDNA, the genomic sequence of our isolate was deposited in the NCBI GenBank database.

### 4.4. Preparation of Aspergillus niger Inoculum

Liquid formulations of *A. niger* were prepared by growing the fungus in potato dextrose broth. Spore suspension was obtained by mixing vigorously, which was measured using direct microscopic counting with a hemocytometer [92]. The fungal spore suspension used as inoculum was adjusted at a 10^7^-spore mL^−1^ concentration.

### 4.5. Compost Preparation

Compost was prepared following the entire procedure described by Idrovo-Novillo et al. [93] with a slight modification using whole plants as raw material instead of plant shoots. Twenty-five kg of *Pelargonium graveolens* waste material was well mixed with rice straw bulking material (0.5 kg) to provide some free air pores within the composted material required to maintain the aerobic conditions and potassium humate (0.5 kg) in addition to cattle dung (12 kg) and green *Trifolium alexandrinum* L. plants (12 kg) as a source of N element. All these components were well blended, which composed the compost to a respective extent of 50, 0.5, 0.5, 24, and 24%, respectively. The components were then composted in a pilot plant using a turning-pile technique in a pile measuring 20 m in length, 2.5 m in width, and 1.5 m in height. The composting mixture pile was turned over three times a month during the bio-oxidation stage using a front-end-loader tractor and regularly sprinkled with water to maintain about 60% (*v*/*w*) moisture level. The composting process lasted from mid-April to mid-July, up to the combined maturation of all compost materials. The physicochemical properties of the prepared compost used in this experiment are presented in Table 7.

### 4.6. Treatments, Experimental Design, and Agronomic Management

The experimental treatments were laid out in the field as a completely randomized block design (CRBD) replicated thrice. Six treatments were applied in both normal and calcareous soils, for a total of twelve treatments. The treatments applied in this experiment are bio-organo-mineral applications as follows: (1) PK_100%_: the soil received the conventional full recommended dose (FRD) of mineral phosphorus (P) and potassium (K) fertilizers; (2) PK_100%_+C-AN: the soil received PK_100%_ + compost at a rate of 20 t ha^−1^ + PK-solubilizing *A. niger*; (3) PK_75%_+C-AN: the soil received 75% out of conventional FRD of mineral P and K fertilizers + compost at a rate of 20 t ha^−1^ + PK-solubilizing *A. niger*; (4) PK_50%_+C-AN: the soil received 50% out of conventional FRD of mineral P and K fertilizers + compost at a rate of 20 t ha^−1^ + PK-solubilizing *A. niger*; (5) PK_25%_+C-AN: the soil received 25% out of conventional FRD of mineral P and K fertilizers + compost at a rate of 20 t ha^−1^ + PK-solubilizing *A. niger*; (6) PK_0%_+C-AN: neither mineral P nor mineral K fertilizer was added to the tested soil but it only received compost at a rate of 20 t ha^−1^+ PK-solubilizing *A. niger*. For the P fertilizer, the FRD (72 kg P_2_O_5_ ha^−1^) was added basely as calcium superphosphate (12% P_2_O_5_) at planting. For the K fertilizer, the FRD (60 kg K_2_O ha^−1^) as potassium sulfate (48% K_2_O) was applied with 2/3 as a basal dose at planting, and the remaining 1/3 was topdressed at the initiation stage of the main panicle. For the nitrogen (N) fertilizer, 60 kg N ha^−1^ in the form of ammonium nitrate (33.5 N) was added in three equal splits (1/3 at planting and the remaining 2/3 in two additions, 1/3 for each, during the vegetative growth stage before the beginning of panicle emergence). The compost, as an organic amendment, with a rate of 20 t ha^−1^ (local recommendation rate), was thoroughly incorporated and uniformly mixed into the soil (20 cm depth) a week before the sowing for all treated plots using a manual harrow. The root rhizosphere zone of quinoa for each plant hole was drenched twice with 50 mL (25 mL for each time) of freshly prepared *A. niger* spore suspension (10^7^ spores mL^−1^) at sowing time and after 21 days from sowing (DFS).

The quinoa (*Chenopodium quinoa* Willd) cultivar Misr-1 (kindly provided by Crops Research Institute, Ministry of Agriculture and Land Reclamation, Egypt) was chosen for this experiment because it grows promptly and produces great productivity. The sowing dates of quinoa seeds in this experiment were 23 November 2021 (cropping season 2021/22) and 28 November 2022 (cropping season 2022/23) and the harvested dates were 31 March 2022 and 6 April 2023, respectively. About 4–6 quinoa seeds were sown on one side of the ridges at a depth of 1–2 cm in holes 0.2 m apart, which thinned later into two healthy and uniform seedlings per plant hole. The quinoa thinning process was performed at BBCH 12 stage (four true leaves) according to the scale of Sosa-Zuniga et al. [94] guided by the Biologische Bundesanstalt, Bundessortenamt und CHemische Industrie (BBCH) coding scale proposed by Meier et al. [95]. The net area of each experimental plot was 18 m^2^ (3.6 m × 5 m) and included six ridges, 0.6 m wide, 5 m long, and 0.15 m in height. In this experiment, a surface flood irrigation system with fresh irrigation water having an electrical conductivity of 1.58 dS m^−1^, on average over both seasons, was used to regular irrigate quinoa plants on a needed basis. Following the Egyptian Agricultural Research Center’s recommendations, the proper agronomic practices for local commercial quinoa crop production were performed as needed.

### 4.7. Sampling and Measurements

At flowering initiation (BBCH 59/60) stage, according to Sosa-Zuniga et al. [94] and Meier et al. [95], thirty-six fully expanded young leaves were collected randomly from 18 plants from each treatment (6 plants per replicate) for the subsequent physio-biochemical analyses.

#### 4.7.1. Photosynthetic Pigment Assay

The chlorophyll *a* (Chl *a*) and chlorophyll *b* (Chl *b*), and total carotenoid content of quinoa leaves were extracted and quantified using the N, N-dimethyl formamide (DMF) solvent following the protocol of Lichtenthaler and Buschmann [96]. Three leaf discs with an area of 58.91 mm^2^ were submerged in 2 mL DMF (99.8% purity) solvent (Sigma-Aldrich, St. Louis, MO, USA) and stored for 24 h at 4 °C in a dark place. The supernatant after that was separated and its absorbance was read spectrophotometrically at 664, 647, and 480 nm wavelengths for quantification of Chl *a*, Chl *b*, and carotenoid contents, respectively, using a double-beam VWR 6300 UV–Vis spectrophotometer (Palo Alto, CA, USA). The total chlorophyll (Total chl; mg cm^−2^) content was computed by adding Chl *a* to Chl *b* [97], while total photosynthetic pigment (TPP; mg cm^−2^) was calculated by totalizing the Chl *a*, Chl *b*, and carotenoid contents.

#### 4.7.2. Osmoprotectants, Nonenzymatic Antioxidants, and 2,2-diphenyl-1-picrylhydrazyl-Scavenging Activity (DPPH-SA)

Total soluble proteins (TSPs) as mg g^−1^ dry weight (DW) of quinoa leaf tissues was measured following the Lowry–Folin method [98]. Total soluble sugars (TSSs; mg g^−1^ DW) content was measured spectrophotometrically following the anthrone sulfuric acid method described by Fales [99] and Schlegel [100]. Free proline (FPro; mg g^−1^ DW) content was measured following the acidic ninhydrin colorimetry method described by Ábrahám et al. [101] using toluene as a blank solution and L-proline in plotting the standard curve. The reduced glutathione (rGSH; mg g^−1^ DW) content was quantified based on GSH oxidation using Ellman’s reagent (i.e., 5,5′-dithiobis-2-nitrobenzoic acid) according to Anderson’s [102] method. The rGSH concentrations in the samples were then computed using a standard calibration curve obtained from a series of pure GSH concentrations (Merck, Darmstadt, Germany). Ascorbic acid (AsA; mg g^−1^ DW) was extracted and assessed spectrophotometrically at 760 nm as per Jagota and Dani [103] using a Trichloroacetic acid (5% by volume) solution and a Folin–Ciocalteu reagent solution diluted 10-fold (1:10 *v*:*v*). A standard calibration curve using pure AsA was used to determine the AsA content in the samples. Total phenolics (TPs; mg g^−1^ DW) were extracted from dried quinoa leaves in 70% ethanol (*v*/*v*) at 40 °C overnight and quantified as per the colorimetric method adopted by Sauvesty et al. [104], and the TPs concentrations in the samples were computed using a pyrogallol standard calibration curve. The DPPH-SA (%), which is commonly employed to reflect the overall free radical scavenging capacity of nonenzyme antioxidants, was assessed following a modified version of the procedure given by Abe et al. [105].

#### 4.7.3. Determination of Macro- and Micronutrient Contents in Quinoa’s Leaf and Seed

After drying and ground into a fine powdering, the quinoa’s leaf and seed samples were wet digested in concentrated H_2_SO_4_ mixed with HClO_4_ (3:1, *v*/*v*, respectively) by heating up to 280 °C until the plant material was colorless to determine their content of nutrient elements. The total N content (mg g^−1^ DW) of leaf was determined following the Kjeldahl procedure [106], using Gerhardt’s micro Kjeldahl apparatus. The total P content in leaf (mg g^−1^ DW) or in seed (g 100^−1^ g DW) was determined following the molybdenum-reduced molybdophosphoric blue colorimetric procedure [107]. The total content of cationic and trace elements (i.e., potassium—K^+^; sodium—Na^+^; magnesium—Mg^2+^; calcium—Ca^2+^; iron—Fe^2+^;zinc—Zn^2+^; or manganese—Mn^2+^) in quinoa leaves (mg g^−1^ DW) or seeds (g 100^−1^ g DW) was determined [108] using flame atomic absorption spectroscopy (Spectra-55 AA, Agilent Technologies, CA, USA).

#### 4.7.4. Quinoa Seed’s Proximate Chemical Composition

Following the Association of Official Analytical Chemists (AOAC) standard methodologies [109], the proximate chemical composition of raw seeds of quinoa was determined. The moisture content in a 5 g crushed seed sample of quinoa was assessed using the AOAC 934.01 method with an electric oven at 105 °C until constant weight. The ash content (incineration in muffle furnace at 550 °C until constant weight), lipids (extraction with Merck petroleum ether has b.p. 40–60 °C using Soxhlet extractor for 6 h), and crude protein (Kjeldahl digestion with the factor of 6.25 for conversion of the total N content to protein) were determined according to the AOAC methods of 923.03, 920.39C, and 979.09A, respectively. Total dietary fiber content was measured using the AOAC 978.10 method. The following formula was used to compute the carbohydrates percentage: carbohydrates (%) = 100 − moisture (%) + ash (%) + protein (%) + lipids (%) + dietary fiber (%).

#### 4.7.5. Quinoa Seed’s Phytochemical and Antioxidant Activity

For quinoa seed extract preparation, a crushed seed sample with mass around 10 g was homogenized with 50 mL 80% ethanol. The mixture was placed in agitation overnight at 160 rpm in a shaker. Then, the homogenate was filtered, and the supernatant was removed. Total phenolic content (TPC) was determined using Folin–Ciocalteu’s reagent as outlined by Singleton et al. [110]. The liquid extract was diluted and mixed with Folin–Ciocalteu’s reagent and 20% aqueous Na_2_CO_3_ solution, then incubated in the dark for 1 h at room temperature (25 °C). After incubation, the absorbance of the mixture was measured at 765 nm using a spectronic 2000 spectrophotometer. The TPC was expressed as mg gallic acid equivalent per 100 g on a dry weight basis. For total flavonoid content (TFC) determination, briefly, one ml aliquot of extract was mixed with one ml 10% aqueous AlCl_3_ solution. The mixture was incubated in the dark for 1 h at room temperature (25 °C). Absorbance of the final mixture was determined at 510 nm against a blank reaction. The TFC was expressed as mg quercetin equivalent per 100 g on a dry weight basis.

For assaying the total DPPH-SA, four aliquots (25, 50, 75, and 100 μL) of extract were added to 3.9 mL of methanolic solution of DPPH radical. The mixture was agitated and then kept in the absence of light for 30 min and the absorbance was determined at 517 nm using a spectronic 2000 spectrophotometer. The total DPPH-SA was calculated according to the following equation [111]: Total DPPH-SA (%) = [(Abs_blank_ − Abs_sample_)/Abs_blank_] × 100; where Abs_blank_ = absorbance of DPPH radical + methanol at 0 min and Abs_sample_ = absorbance of DPPH radical + sample at 30 min.

The concentration of extract that corresponding to a 50% inhibition of DPPH^•^ radical, which is expressed as the half maximal inhibitory concentration (IC_50_), was obtained from the graph plotted between the inhibition percentages and the extract concentrations [112]. The antiradical power (ARP) was determined as the efficient concentration IC_50’_s reciprocal value and calculated as follows: 1/IC_50_.

#### 4.7.6. Soil Microbial Evaluation

At the initial and end of the experiment, soil samples of the rhizosphere zone at 0–20 cm depth of each experimental plot from the normal and calcareous soils, totaling thirty-six samples, were collected using a soil auger of 5 cm diameter. Each sample was 10-fold serially diluted up to 10^−5^ dilutions and the counts of the phosphorus-solubilizing microorganisms (PSMs) and *Azotobacter* bacteria were determined, expressed as colony-forming units per gram soil (cfu g^−1^ soil). The count of PSMs was determined by allowing microorganisms in the soil samples to grow on Pikovskaya’s selective media for 7 to 10 days at a temperature of 25 °C. Colonies rounded by clear halo zone were detected and the number of cfu g^−1^ soil was counted. Serial dilution pour plate technique was used to count *Azotobacter* sp. using Ashby’s N-free agar medium containing 0.5 g K_2_HPO_4_, 0.2 g MgSO_4_, 0.2 g NaCl, 5 g CaCO_3_, 10 g sucrose, 12 g agar, 1000 mL distilled H_2_O, and traces of manganese, iron, and molybdenum elements. The number of *Azotobacter* sp. as a cfu g^−1^ soil was counted after the formation of medium–large, moist colonies during the incubation for 48–72 h at a temperature of 28 °C.

#### 4.7.7. Quinoa’s Growth, Yield and Yield-Related Attributes, and Harvest Index

At the seed ripening (BBCH 89/90) stage, ten individual quinoa plants were randomly sampled from each experimental plot. These individual plants were utilized to measure plant height (in cm) from the base of the main shoot to the tip of uppermost shoot using a meter scale and plant dry weight (in g) by oven-drying at 70 ± 3 °C to constant weight for dry matter estimation. All the quinoa plants from a 12 m^2^ area representing the four central ridges of each experimental plot to avoid edge effects were harvested, sun-dried for three days at the field, and weighed for biological yield (in t ha^−1^) estimation. Quinoa seeds were threshed, cleansed, and weighed to quantify the seed yield (in t ha^−1^). For moisture determination, a 150 g representative seed sample was oven-dried at 105 °C for 24 h until a constant weight was attained and then weighed once more to determine the adjusted seed yield based on 12% moisture. Seed harvest index (HI) percentage was calculated as the following equation: HI (%) = [seed yield (t ha^−1^) based on 12% moisture/biological yield (t ha^−1^)] × 100. The hectoliter weight of quinoa seeds expressed as kg hL^−1^ was determined by placing the seeds in a 250 mL cylindrical vessel and weighed, and the weight was multiplied by four hundred to obtain the kg weight of seeds per 100 L.

### 4.8. Statistical Analysis

Microsoft Excel^®^ 2016 spreadsheet was utilized to compute means ± 1 standard error (SE) and data visualization. The normality assumption for all obtained data in each season was verified using the Shapiro–Wilk numerical normality test. Data that had a normal distribution were statistically analyzed using a two-way analysis of variance (ANOVA) followed by Duncan’s test (*p* ≤ 0.05 * or *p* ≤ 0.01 **). Before performing the combined ANOVA [113], a homogeneity test of the two-season error variances of all variables was assessed. The outputs of the homogeneity test for all measurements exhibited statistical validity to perform further combined ANOVA (Tables S1–S4), using 11th edition Genstat software (VSN International Ltd., Oxford, UK) as per Casella [114]. The soil type, bio-organo-mineral treatments, and their interaction were considered fixed factors, while replications, cropping seasons, and their interaction were considered random factors. The PCA biplot and Pearson’s correlogram among the selected variables were performed by using the statistical R (version 4.0.2) software package.

## 5. Conclusions

It can be concluded that co-application of compost with *Aspergillus niger* could help to reduce phosphorus and potassium mineral fertilization to a notable extent without limiting yield as well as mitigate the deleterious influence of calcareous soil on quinoa plants. Furthermore, the combined use of *Aspergillus niger* and compost with reduced phosphorus and potassium fertilization resulted in elevated osmolytes and antioxidant accumulation, and nutrient acquisition, subsequently promoting the chlorophylls and carotenoid contents, as well as increasing seed yield and quality of quinoa plants grown in calcareous soil conditions. In calcareous soil, the application of bioorganic fertilizer enhanced the population of P-solubilizing microorganisms, whereas in normal soil, PK mineral fertilization with bioorganic fertilizers increased the *Azotobacter* sp. population. Future research should focus on employing direct measures of enzyme activity related to phosphorus/potassium solubilization since this will provide a deep understanding of different pathways connected to the beneficial effects of *Aspergillus niger* inoculation.

## Figures and Tables

**Table 1:** Interactive effect of soil type (ST) and compost with phosphate (P)–potassium (K)-solubilizing *Aspergillus niger* (PK+C-AN) level on leaf chlorophylls, and carotenoids of quinoa grown in (SI) 2021/22 and (SII) 2022/23 winter seasons.

ST	PK+C-AN	Chl *a*	Chl *b*	Total Chl	Carotenoids	TPP
		(mg cm^−2^)				
Nor	PK_100%_	27.0 ± 0.4 cd	5.39 ± 0.27 a	32.4 ± 0.4 e	9.03 ± 0.16 ab	41.4 ± 0.5 de
	PK_100%_+C-AN	34.3 ± 0.4 a	7.64 ± 0.29 a	41.9 ± 0.5 a	9.48 ± 0.28 a	51.4 ± 0.7 a
	PK_75%_+C-AN	28.7 ± 0.6 c	7.37 ± 0.16 a	36.0 ± 0.7 bc	8.57 ± 0.08 bc	44.6 ± 0.7 bc
	PK_50%_+C-AN	27.6 ± 0.5 c	7.15 ± 0.19 a	34.8 ± 0.6 cd	8.41 ± 0.15 c	43.2 ± 0.7 cd
	PK_25%_+C-AN	27.0 ± 0.5 cd	6.93 ± 0.11 a	33.9 ± 0.6 de	8.51 ± 0.17 c	42.4 ± 0.5 d
	PK_0%_+C-AN	22.5 ± 0.3 f	6.01 ± 0.60 a	28.5 ± 0.4 fg	7.92 ± 0.13 d	36.4 ± 0.3 fg
Calc	PK_100%_	23.6 ± 0.7 ef	4.61 ± 0.36 a	28.2 ± 0.4 g	7.13 ± 0.38 fg	35.3 ± 0.8 gh
	PK_100%_+C-AN	30.6 ± 1.2 b	6.80 ± 0.31 a	37.4 ± 1.0 b	9.01 ± 0.17 ab	46.5 ± 1.1 b
	PK_75%_+C-AN	28.7 ± 0.8 c	5.74 ± 0.21 a	34.4 ± 0.8 cd	8.44 ± 0.14 c	42.9 ± 0.8 cd
	PK_50%_+C-AN	27.0 ± 0.8 cd	5.25 ± 0.13 a	32.2 ± 0.8 e	7.64 ± 0.11 de	39.9 ± 0.8 e
	PK_25%_+C-AN	25.2 ± 0.5 de	4.96 ± 0.11 a	30.2 ± 0.5 f	7.36 ± 0.16 ef	37.6 ± 0.4 f
	PK_0%_+C-AN	23.0 ± 0.3 f	4.56 ± 0.14 a	27.5 ± 0.4 g	6.67 ± 0.17 g	34.2 ± 0.3 h
*p*-value for ST × PK+C-AN		0.007 **	0.073 ^ns^	0.047 *	<0.001 **	0.016 *

Values are means ± standard error (*n* = 3). * and ** indicate differences at *p* ≤ 0.05 and *p* ≤ 0.01 probability level, respectively. ^ns^ = no significant difference. Mean values for each factor followed by the same lowercase letter in each column are not significantly different according to the Duncan test (*p* ≤ 0.05). PK_100%_ = 72 kg P_2_O_5_ ha^−1^ + 60 kg K_2_O ha^−1^, PK_75%_ = 54 kg P_2_O_5_ ha^−1^ + 45 kg K_2_O ha^−1^, PK_50%_ = 36 kg P_2_O_5_ ha^−1^ + 30 kg K_2_O ha^−1^, PK_25%_ = 18 kg P_2_O_5_ ha^−1^ + 15 kg K_2_O ha^−1^, PK_0%_ = 0 kg P_2_O_5_ ha^−1^ + 0 kg K_2_O ha^−1^, and compost was added with a rate of 20 t ha^−1^. Normal (Nor), calcareous (Calc), chlorophyll *a* (Chl *a*), chlorophyll *b* (Chl *b*), total chlorophyll (Total Chl), and total photosynthetic pigments (TPP).

**Table 2:** Interactive effect of soil type (ST) and compost with phosphate (P)–potassium (K)-solubilizing *Aspergillus niger* (PK+C-AN) level on proximate chemical composition of quinoa’s seeds grown in (S_I_) 2021/22 and (S_II_) 2022/23 winter seasons.

ST	PK+C-AN	Crude Protein	Ash	Crude Lipid	Crude Fiber	Carbohydrates
		(%)				
Nor	PK_100%_	14.7 ± 0.10 f	2.61 ± 0.03 e	6.35 ± 0.15 a–c	3.57 ± 0.04 f	64.5 ± 0.44 f
	PK_100%_+C-AN	16.1 ± 0.12 a	3.20 ± 0.07 c	6.48 ± 0.15 ab	3.61 ± 0.04 e	62.4 ± 0.52 i
	PK_75%_+C-AN	15.2 ± 0.11 d	2.91 ± 0.06 d	6.55 ± 0.15 ab	3.70 ± 0.04 d	63.9 ± 0.46 g
	PK_50%_+C-AN	15.4 ± 0.11 c	2.97 ± 0.04 d	6.45 ± 0.22 ab	3.82 ± 0.04 c	63.1 ± 0.47 h
	PK_25%_+C-AN	15.6 ± 0.11 b	2.41 ± 0.03 f	6.59 ± 0.25 a	4.00 ± 0.04 b	63.3 ± 0.47 h
	PK_0%_+C-AN	14.9 ± 0.10 e	1.48 ± 0.10 h	6.13 ± 0.15 c–e	4.13 ± 0.05 a	65.2 ± 0.43 e
Calc	PK_100%_	11.9 ± 0.08 k	2.00 ± 0.03 g	5.90 ± 0.14 e	3.24 ± 0.04 l	71.1 ± 0.35 a
	PK_100%_+C-AN	12.4 ± 0.13 j	3.67 ± 0.04 a	6.03 ± 0.14 de	3.27 ± 0.04 k	68.5 ± 0.40 b
	PK_75%_+C-AN	13.3 ± 0.11 i	3.34 ± 0.04 b	6.03 ± 0.14 de	3.36 ± 0.04 j	67.7 ± 0.39 cd
	PK_50%_+C-AN	13.4 ± 0.09 i	2.94 ± 0.03 d	5.87 ± 0.15 e	3.40 ± 0.04 i	68.1 ± 0.37 bc
	PK_25%_+C-AN	13.6 ± 0.10 h	2.60 ± 0.06 e	6.15 ± 0.15 c–e	3.74 ± 0.04 h	68.3 ± 0.37 b
	PK_0%_+C-AN	14.0 ± 0.10 g	2.57 ± 0.05 e	6.28 ± 0.15 b–d	3.52 ± 0.04 g	67.6 ± 0.39 d
*p*-value for ST × PK+C-AN		<0.001 **	<0.001 **	0.003 **	<0.001 **	<0.001 **

Values are means ± standard error (*n* = 3). ** indicates difference at *p* ≤ 0.01 probability level. Mean values for each factor followed by the same lowercase letter in each column are not significantly different according to the Duncan test (*p* ≤ 0.05). PK_100%_ = 72 kg P_2_O_5_ ha^−1^ + 60 kg K_2_O ha^−1^, PK_75%_ = 54 kg P_2_O_5_ ha^−1^ + 45 kg K_2_O ha^−1^, PK_50%_ = 36 kg P_2_O_5_ ha^−1^ + 30 kg K_2_O ha^−1^, PK_25%_ = 18 kg P_2_O_5_ ha^−1^ + 15 kg K_2_O ha^−1^, PK_0%_ = 0 kg P_2_O_5_ ha^−1^ + 0 kg K_2_O ha^−1^, and compost was added with a rate of 20 t ha^−1^. Normal (Nor) and calcareous (Calc).

**Table 3:** Interactive effect of soil type (ST) and compost with phosphate (P)–potassium (K)-solubilizing *Aspergillus niger* (PK+C-AN) level on phytochemicals and antioxidant activity of quinoa’s seeds grown in (S_I_) 2021/22 and (S_II_) 2022/23 winter seasons.

ST	PK+C-AN	TPC	TFC	IC50 (mg mL^−1^)	ARP
		(mg 100^−1^ g Dry Seed)			
Nor	PK_100%_	66.0 ± 0.53 f	26.2 ± 0.38 f	0.43 ± 0.00 e	2.08 ± 0.02 g
	PK_100%_+C-AN	84.0 ± 0.56 a	44.5 ± 0.41 a	0.54 ± 0.01 a	2.78 ± 0.05 c
	PK_75%_+C-AN	74.9 ± 0.50 c	39.6 ± 0.41 b	0.50 ± 0.01 b	2.40 ± 0.04 d
	PK_50%_+C-AN	75.4 ± 0.52 c	33.7 ± 0.41 d	0.47 ± 0.01 c	2.42 ± 0.03 d
	PK_25%_+C-AN	69.9 ± 0.52 d	30.4 ± 0.32 e	0.43 ± 0.01 e	2.21 ± 0.03 ef
	PK_0%_+C-AN	53.6 ± 0.36 i	30.0 ± 0.28 e	0.37 ± 0.01 f	1.91 ± 0.03 h
Calc	PK_100%_	57.1 ± 0.43 h	23.6 ± 0.55 g	0.35 ± 0.00 g	2.28 ± 0.02 e
	PK_100%_+C-AN	77.7 ± 0.52 b	43.6 ± 0.44 a	0.48 ± 0.01 c	3.21 ± 0.03 a
	PK_75%_+C-AN	68.3 ± 1.24 e	36.0 ± 1.12 c	0.45 ± 0.00 d	2.94 ± 0.03 b
	PK_50%_+C-AN	69.2 ± 0.72 de	30.0 ± 0.27 e	0.43 ± 0.00 e	2.75 ± 0.03 c
	PK_25%_+C-AN	63.6 ± 0.43 g	25.1 ± 0.30 f	0.38 ± 0.00 f	2.42 ± 0.03 d
	PK_0%_+C-AN	51.5 ± 0.35 j	21.9 ± 0.27 h	0.32 ± 0.00 h	2.16 ± 0.02 f
*p*-value for ST × PK+C-AN		<0.001 **	<0.001 **	0.005 **	<0.001 **

Values are means ± standard error (*n* = 3). ** indicates difference at *p* ≤ 0.01 probability level. Mean values for each factor followed by the same lowercase letter in each column are not significantly different according to the Duncan test (*p* ≤ 0.05). PK_100%_ = 72 kg P_2_O_5_ ha^−1^ + 60 kg K_2_O ha^−1^, PK_75%_ = 54 kg P_2_O_5_ ha^−1^ + 45 kg K_2_O ha^−1^, PK_50%_ = 36 kg P_2_O_5_ ha^−1^ + 30 kg K_2_O ha^−1^, PK_25%_ = 18 kg P_2_O_5_ ha^−1^ + 15 kg K_2_O ha^−1^, PK_0%_ = 0 kg P_2_O_5_ ha^−1^ + 0 kg K_2_O ha^−1^, and compost was added with a rate of 20 t ha^−1^. Normal (Nor), calcareous (Calc), total phenolic compounds (TPC), total flavonoid compounds (TFC), half maximal inhibitory concentration (IC_50_), and antiradical power (ARP).

**Table 4:** Interactive effect of soil type (ST) and compost with phosphate (P)–potassium (K)-solubilizing *Aspergillus niger* (PK+C-AN) level on seed mineral (i.e., phosphorus—P; potassium—K^+^; calcium—Ca^2+^; magnesium—Mg^2+^, sodium—Na^2+^, iron—Fe^2+^, and zinc—Zn^2+^) contents of quinoa grown in (S_I_) 2021/22 and (S_II_) 2022/23 winter seasons.

ST	PK+C-AN	P	K^+^	Ca^2+^	Mg^2+^	Na^+^	Fe^2+^	Zn^2+^
		(g 100^−1^ g Dry Seed)						
Nor	PK_100%_	0.35 ± 0.00 ef	2.52 ± 0.02 g	1.63 ± 0.02 a	0.84 ± 0.05 e	0.30 ± 0.01 c	0.92 ± 0.02 h	0.46 ± 0.01 e
	PK_100%_+C-AN	0.48 ± 0.01 a	3.19 ± 0.11 a	1.95 ± 0.04 a	1.42 ± 0.12 a	0.37 ± 0.02 a	1.15 ± 0.08 a	0.50 ± 0.01 a
	PK_75%_+C-AN	0.44 ± 0.01 b	2.70 ± 0.03 c	1.74 ± 0.02 a	1.08 ± 0.12 b	0.34 ± 0.01 b	1.01 ± 0.02 c	0.49 ± 0.01 b
	PK_50%_+C-AN	0.42 ± 0.00 c	2.64 ± 0.02 e	1.69 ± 0.02 a	0.87 ± 0.07 c	0.30 ± 0.00 c	0.95 ± 0.00 f	0.47 ± 0.01 c
	PK_25%_+C-AN	0.33 ± 0.00 f	2.59 ± 0.00 f	1.49 ± 0.03 a	0.84 ± 0.07 de	0.29 ± 0.00 d	0.89 ± 0.01 i	0.47 ± 0.01 c
	PK_0%_+C-AN	0.27 ± 0.01 h	2.30 ± 0.04 i	1.23 ± 0.02 a	0.63 ± 0.01 k	0.25 ± 0.01 g	0.86 ± 0.01 j	0.43 ± 0.00 g
Calc	PK_100%_	0.36 ± 0.01 e	2.21 ± 0.02 k	1.42 ± 0.02 a	0.68 ± 0.04 i	0.21 ± 0.02 j	0.82 ± 0.01 k	0.45 ± 0.00 f
	PK_100%_+C-AN	0.47 ± 0.00 a	2.89 ± 0.01 b	1.72 ± 0.03 a	0.85 ± 0.04 d	0.28 ± 0.01 e	1.08 ± 0.04 b	0.47 ± 0.00 c
	PK_75%_+C-AN	0.42 ± 0.01 c	2.69 ± 0.08 d	1.55 ± 0.02 a	0.80 ± 0.05 f	0.26 ± 0.00 f	0.98 ± 0.03 d	0.46 ± 0.00 d
	PK_50%_+C-AN	0.40 ± 0.01 d	2.47 ± 0.03 h	1.52 ± 0.02 a	0.75 ± 0.02 g	0.24 ± 0.01 h	0.96 ± 0.01 e	0.45 ± 0.00 ef
	PK_25%_+C-AN	0.30 ± 0.01 g	2.22 ± 0.03 j	1.38 ± 0.02 a	0.70 ± 0.02 h	0.22 ± 0.01 i	0.94 ± 0.01 g	0.46 ± 0.01 e
	PK_0%_+C-AN	0.23 ± 0.01 i	2.20 ± 0.00 k	1.03 ± 0.02 a	0.66 ± 0.02 j	0.16 ± 0.03 k	0.82 ± 0.00 k	0.44 ± 0.01 g
*p*-value for ST × PK+C-AN		<0.001 **	<0.001 **	0.220 ^ns^	<0.001 **	<0.001 **	<0.001 **	<0.001 **

Values are means ± standard error (*n* = 3). ** indicates difference at *p* ≤ 0.01 probability level. ^ns^ = no significant difference. Mean values for each factor followed by the same lowercase letter in each column are not significantly different according to the Duncan test (*p* ≤ 0.05). PK_100%_ = 72 kg P_2_O_5_ ha^−1^ + 60 kg K_2_O ha^−1^, PK_75%_ = 54 kg P_2_O_5_ ha^−1^ + 45 kg K_2_O ha^−1^, PK_50%_ = 36 kg P_2_O_5_ ha^−1^ + 30 kg K_2_O ha^−1^, PK_25%_ = 18 kg P_2_O_5_ ha^−1^ + 15 kg K_2_O ha^−1^, PK_0%_ = 0 kg P_2_O_5_ ha^−1^ + 0 kg K_2_O ha^−1^, and compost was added with a rate of 20 t ha^−1^. Normal (Nor) and calcareous (Calc).

**Table 5:** Effect of soil type, compost with phosphate (P)–potassium (K)-solubilizing *Aspergillus niger* (PK+C-AN) level, and their interaction on yield and yield-related attributes of quinoa grown in (S_I_) 2021/22 and (S_II_) 2022/23 winter seasons.

ST	PK+C-AN	Plant Height (cm)	Plant Dry Weight (g)	HW (kg hL^−1^)	Seed Yield	Biological Yield	HI (%)
					(t ha^−1^)		
Nor	PK_100%_	96.8 ± 1.3 d	26.5 ± 0.2 c	67.6 ± 0.4 fg	2.24 ± 0.04 e	4.95 ± 0.24 d	45.7 ± 1.9 a
	PK_100%_+C-AN	112.5 ± 1.3 a	39.3 ± 0.4 a	70.1 ± 0.5 b	3.27 ± 0.05 a	7.70 ± 0.20 a	42.6 ± 1.8 a
	PK_75%_+C-AN	109.8 ± 1.0 b	29.9 ± 0.8 b	69.3 ± 0.5 b–d	3.20 ± 0.04 a	6.96 ± 0.26 b	46.1 ± 1.4 a
	PK_50%_ + C-AN	108.8 ± 0.7 b	26.5 ± 0.2 c	68.5 ± 0.5 d–f	3.01 ± 0.06 b	6.64 ± 0.43 b	46.3 ± 3.2 a
	PK_25%_+C-AN	107.3 ± 0.8 bc	24.2 ± 0.4 d	68.2 ± 0.4 e–g	2.01 ± 0.09 f	5.42 ± 0.25 c	37.4 ± 1.8 a
	PK_0%_+C-AN	104.8 ± 1.0 c	23.7 ± 0.5 d	66.5 ± 0.5 h	1.76 ± 0.05 g	4.21 ± 0.27 ef	42.5 ± 1.9 a
Calc	PK_100%_	80.3 ± 1.0 g	13.0 ± 0.4 i	67.6 ± 0.6f g	1.57 ± 0.04 h	3.01 ± 0.04 g	52.3 ± 1.5 a
	PK_100%_+C-AN	93.0 ± 1.4 e	21.2 ± 0.5 e	72.4 ± 1.03 a	2.50 ± 0.07 c	4.79 ± 0.04 d	52.2 ± 1.0 a
	PK_75%_+C-AN	91.8 ± 1.3 e	19.0 ± 0.2 f	69.4 ± 0.1 bc	2.43 ± 0.07 cd	4.56 ± 0.10 de	53.3 ± 0.8 a
	PK_50%_+C-AN	90.8 ± 1.5 ef	17.2 ± 0.4 g	69.0 ± 0.5 c–e	2.27 ± 0.07 de	4.25 ± 0.11 ef	53.7 ± 1.8 a
	PK_25%_+C-AN	88.3 ± 1.8 f	16.2 ± 0.3 h	68.4 ± 0.4 d–f	2.12 ± 0.15 ef	4.02 ± 0.19 f	52.4 ± 1.3 a
	PK_0%_+C-AN	82.2 ± 1.0 g	12.4 ± 0.2 i	67.4 ± 0.2 g	1.63 ± 0.09 gh	3.18 ± 0.10 g	51.3 ± 2.8 a
*p*-value for ST × PK+C-AN		0.034 *	<0.001 **	0.002 **	<0.001 **	<0.001 **	0.077 ^ns^

Values are means ± standard error (*n* = 3). * and ** indicate differences at *p* ≤ 0.05 and *p* ≤ 0.01 probability level, respectively. ^ns^ = no significant difference. Mean values for each factor followed by the same lowercase letter in each column are not significantly different according to the Duncan test (*p* ≤ 0.05). PK_100%_ = 72 kg P_2_O_5_ ha^−1^ + 60 kg K_2_O ha^−1^, PK_75%_ = 54 kg P_2_O_5_ ha^−1^ + 45 kg K_2_O ha^−1^, PK_50%_ = 36 kg P_2_O_5_ ha^−1^ + 30 kg K_2_O ha^−1^, PK_25%_ = 18 kg P_2_O_5_ ha^−1^ + 15 kg K_2_O ha^−1^, PK_0%_ = 0 kg P_2_O_5_ ha^−1^ + 0 kg K_2_O ha^−1^, and compost was added with a rate of 20 t ha^−1^. Hectoliter weight (HW) and harvest index (HI).

**Table 6:** Initial physicochemical and microbial properties (*n* = 3) for the upper 0.3 m soil horizon of both experimental soils (i.e., normal and calcareous) at pre-sowing over both 2021/22 and (S_II_) 2022/23 winter seasons.

Property	Unite	Soil Type	
		Normal	Calcareous
Sand	(%)	79.90 ± 0.72	79.00 ± 0.91
Silt		9.60 ± 0.51	10.10 ± 0.75
Clay		10.50 ± 0.46	10.90 ± 0.42
Texture class		S.L	S.L
Dry bulk density	(g cm^−3^)	1.65 ± 0.06	1.59 ± 0.04
pH (in 1:2.5 soil: water (*w*:*v*) suspension)		7.52 ± 0.03	7.69 ± 0.05
ECe (in soil past (1:2.5, *w*:*v*) extract)	(dS m^−1^)	3.56 ± 0.05	3.19 ± 0.05
Cation exchange capacity	(meq/100 g soil)	9.35 ± 0.32	9.89 ± 0.45
CaCO_3_	(%)	6.58 ± 0.07	15.54 ± 0.09
Organic carbon		47.09 ± 0.95	43.60 ± 0.81
Organic matter		0.81 ± 0.02	0.75 ± 0.03
** Exchangeable cations			
Ca^2+^	(meq L^−1^)	12.89 ± 0.24	17.66 ± 0.33
Mg^2+^		9.54 ± 0.36	10.59 ± 0.18
Na^+^		11.25 ± 0.18	12.75 ± 0.26
K^+^		0.78 ± 0.01	1.00 ± 0.02
** Exchangeable anions			
Cl^−^	(meq L^−1^)	8.17 ± 0.62	14.21 ± 0.56
SO_4_^2−^		25.07 ± 0.74	21.54 ± 0.66
HCO_3_^−^		1.22 ± 0.03	6.25 ± 0.08
CO_3_^2−^		-	-
Available soil nutrients			
N	(%)	0.08 ± 0.01	0.04 ± 0.01
P	(mg kg^−1^ soil)	7.54 ± 0.10	5.44 ± 0.12
K^+^		50.00 ± 1.4	42.08 ± 1.2
Fe^2+^		2.20 ± 0.05	2.00 ± 0.06
Mg^2+^		4.10 ± 0.22	3.90 ± 0.18
Zn^2+^		0.79 ± 0.03	0.70 ± 0.05
Cu^2+^		0.51 ± 0.05	0.44 ± 0.06
Total microbial count			
PSMs	(cfu × 10^3^ g^−1^ soil)	0.00	0.00
*Azotobacter* sp.		2.00 ± 0.46	0.00

** measured in soil paste for soil analysis (1:2.5 soil: water, *w*/*v*, respectively) extract. Values are means ± standard error (*n* = 3). Sandy loam (S.L); Phosphate-solubilizing microorganisms (PSMs).

**Table 7:** The physicochemical characteristics of compost organic amendment used in this study.

Parameter	Unite	Mean Value ± SE
Bulk density	(g cm^−3^)	0.75 ± 0.09
Moisture content	(%)	38.40 ± 0.93
Water holding pores		25.50 ± 0.65
pH (in 1:2.5 compost: water (*w*:*v*) suspension)		7.64 ± 0.05
ECe	(dS m^−1^)	2.10 ± 0.05
CaCO_3_	(%)	1.65 ± 0.03
Organic carbon		27.06 ± 0.65
Organic matter		46.55 ± 0.70
Carbon/nitrogen ratio		20.81 ± 0.35
Total macro-and micronutrients		
N	(%)	1.30 ± 0.03
P		0.95 ± 0.02
K^+^		0.84 ± 0.01
Fe^+2^	(mg kg^−1^)	0.979 ± 18
Mg^2+^		0.469 ± 26
Zn^2+^		0.659 ± 33

SE= standard error (*n* = 3); EC_e_ = electrical conductivity of the compost past extract.

## Data Availability

The datasets used and/or analyzed during the current study are available from the corresponding author on reasonable request.

## Supplementary Materials

The following supporting information can be downloaded at: https://www.mdpi.com/article/10.3390/plants12173071/s1.

## References

[1] Wassif M.M., Wassif O.M. (2021). Sustainable soil management to mitigate soil erosion hazards in Egypt. Management and Development of Agricultural and Natural Resources in Egypt’s Desert.

[2] Alghamdi S.A., Alharby H.F., Abdelfattah M.A., Mohamed I.A., Hakeem K.R., Rady M.M., Shaaban A. (2023). *Spirulina platensis*-inoculated humified compost boosts rhizosphere soil hydro-physico-chemical properties and *Atriplex nummularia* forage yield and quality in an arid saline calcareous soil. J. Soil Sci. Plant Nutr..

[3] Beheshti M., Alikhani H.A., Pourbabaee A.A., Etesami H., Rahmani H.A., Noroozi M. (2022). Enriching periphyton with phosphate-solubilizing microorganisms improves the growth and concentration of phosphorus and micronutrients of rice plant in calcareous paddy soil. Rhizosphere.

[4] Mirzaee S., Ghorbani-Dashtaki S. (2018). Deriving and evaluating hydraulics and detachment models of rill erosion for some calcareous soils. Catena.

[5] Souei A., Zouaghi T. (2021). Using statistical models and GIS to delimit the groundwater recharge potential areas and to estimate the infiltration rate: A case study of Nadhour-Sisseb-El Alem Basin, Tunisia. J. Arid Land.

[6] Barka H.A.F., Benzaghta M.A., Kasheem A.M. (2018). Effect of different organic matters on chemical properties of calcareous soil. Sirte Univ. Sci. J..

[7] Taalab A.S., Ageeb G.W., Siam H.S., Mahmoud S.A. (2019). Some characteristics of calcareous soils. A review. Middle East J..

[8] FAO (2016). FAO Soils Portal: Management of Calcareous Soils. http://www.fao.org/soils-portal/soil-management/managementof-some-problem-soils/calcareous-soils/ar/.

[9] Mekdad A.A.A., El-Sherif A.M.A., Rady M.M., Shaaban A. (2022). Culture management and application of humic acid in favor of *Helianthus annuus* L. oil yield and nutritional homeostasis in a dry environment. J. Soil Sci. Plant Nutr..

[10] Mahidi S.S., Hassan G.I., Hussain A., Rasool F. (2011). Phosphorus availability issue-Its fixation and role of phosphate solubilizing bacteria in phosphate solubilization-Case study. Res. J. Agric. Sci..

[11] Shaaban A., El-Mageed T.A.A., El-Momen W.R.A., Saudy H.S., Al-Elwany O.A.A.I. (2023). The integrated application of phosphorous and zinc affects the physiological status, yield and quality of canola grown in phosphorus-suffered deficiency saline soil. Gesunde Pflanzen.

[12] Zakirullah M., Khalil S. (2012). Timing and rate of phosphorus application influence maize phenology, yield and profitability in Northwest Pakistan. Int. J. Plant Prod..

[13] Naeem A., Akhtar M., Ahmad W. (2013). Optimizing available phosphorus in calcareous soils fertilized with diammonium phosphate and phosphoric acid using Freundlich adsorption isotherm. Sci. World J..

[14] Rawat P., Das S., Shankhdhar D., Shankhdhar S.C. (2021). Phosphate-solubilizing microorganisms: Mechanism and their role in phosphate solubilization and uptake. J. Soil Sci. Plant Nutr..

[15] Hussain S., Sharif M., Ahmad W. (2021). Selection of efficient phosphorus solubilizing bacteria strains and mycorrhizea for enhanced cereal growth, root microbe status and N and P uptake in alkaline calcareous soil. Soil Sci. Plant Nutr..

[16] Wahid F., Fahad S., Danish S., Adnan M., Yue Z., Saud S., Siddiqui M.H., Brtnicky M., Hammerschmiedt T., Datta R. (2020). Sustainable management with mycorrhizae and phosphate solubilizing bacteria for enhanced phosphorus uptake in calcareous soils. Agriculture.

[17] Li Z., Bai T., Dai L., Wang F., Tao J., Meng S., Hu Y., Wang S., Hu S. (2016). A study of organic acid production in contrasts between two phosphate solubilizing fungi: *Penicillium oxalicum* and *Aspergillus niger*. Sci. Rep..

[18] Tian D., Xia J., Zhou N., Xu M., Li X., Zhang L., Du S., Gao H. (2022). The utilization of phosphogypsum as a sustainable phosphate-based fertilizer by *Aspergillus niger*. Agronomy.

[19] Naeem U., Afzaal M., Qazi A., Yasar A., Mahfooz Y., Naz A.U., Awan H. (2022). Investigating the effect of Aspergillus niger inoculated press mud (biofertilizer) on the potential of enhancing maize (*Zea mays* L.) yield, potassium use efficiency and potassium agronomic efficiency. Cereal Res. Commun..

[20] Abd El-Mageed T.A., Mekdad A.A.A., Rady M.O.A., Abdelbaky A.S., Saudy H.S., Shaaban A. (2022). Physio-biochemical and agronomic changes of two sugar beet cultivars grown in saline soil as influenced by potassium fertilizer. J. Soil Sci. Plant Nutr..

[21] Attia M.M. (2019). Status of potassium in some calcareous soils of Egypt and factors affecting its forms. Ann. Agric. Sci. Moshtohor..

[22] Basak B.B., Maity A., Ray P., Biswas D.R., Roy S. (2022). Potassium supply in agriculture through biological potassium fertilizer: A promising and sustainable option for developing countries. Arch. Agron. Soil Sci..

[23] Sattar A., Naveed M., Ali M., Zahir Z.A., Nadeem S.M., Yaseen M., Meena V.S., Farooq M., Singh R., Rahman M. (2019). Perspectives of potassium solubilizing microbes in sustainable food production system: A review. Appl. Soil Ecol..

[24] Kour D., Rana K.L., Yadav A.N., Yadav N., Kumar M., Kumar V., Vyas P., Dhaliwal H.S., Saxena A.K. (2020). Microbial biofertilizers: Bioresources and eco-friendly technologies for agricultural and environmental sustainability. Biocatal. Agric. Biotechnol..

[25] Lian B., Wang B., Pan M., Liu C.Q., Teng H.H. (2008). Microbial release of potassium from K-bearing minerals by thermophilic fungus *Aspergillus fumigatus*. Geochim. Cosmochim. Acta.

[26] Meena V.S., Maurya B.R., Verma J.P. (2014). Does a rhizospheric microorganism enhance K^+^ availability in agricultural soils?. Microbiol. Res..

[27] Pinzari F., Cuadros J., Jungblut A.D., Najorka J., Humphreys-Williams E. (2022). Fungal strategies of potassium extraction from silicates of different resistance as manifested in differential weathering and gene expression. Geochim. Cosmochim. Acta.

[28] Zhang C., Kong F. (2014). Isolation and identification of potassium-solubilizing bacteria from tobacco rhizospheric soil and their effect on tobacco plants. Appl. Soil Ecol..

[29] Qiang X., Ding J., Lin W., Li Q., Xu C., Zheng Q., Li Y. (2019). Alleviation of the detrimental effect of water deficit on wheat (*Triticum aestivum* L.) growth by an indole acetic acid-producing endophytic fungus. Plant Soil.

[30] Sun J., Xu G., Shao H., Xu S. (2012). Potential retention and release capacity of phosphorus in the newly formed wetland soils from the Yellow River Delta, China. Clean–Soil Air Water.

[31] Wang L., Liu L., Zheng B. (2013). Eutrophication development and its key regulating factors in a water-supply reservoir in North China. J. Environ. Sci..

[32] Mundim G.d.S.M., Maciel G.M., Mendes G.d.O. (2022). *Aspergillus niger* as a biological input for improving vegetable seedling production. Microorganisms.

[33] Cihangir N. (2002). Stimulation of the gibberellic acid synthesis by *Aspergillus niger* in submerged culture using a precursor. World J. Microbiol. Biotechnol..

[34] Hung R., Lee Rutgers S. (2016). Applications of Aspergillus in Plant Growth Promotion, New and Future Developments in Microbial Biotechnology and Bioengineering: Aspergillus System Properties and Applications.

[35] Ni H., Wu Y., Zong R., Ren S., Pan D., Yu L., Li J., Qu Z., Wang Q., Zhao G. (2023). Combination of Aspergillus niger MJ1 with Pseudomonas stutzeri DSM4166 or mutant Pseudomonas fluorescens CHA0-nif improved crop quality, soil properties, and microbial communities in barrier soil. Front. Microbiol..

[36] Luo T., Min T., Ru S., Li J. (2022). Response of cotton root growth and rhizosphere soil bacterial communities to the application of acid compost tea in calcareous soil. Appl. Soil Ecol..

[37] Aboukila E.F., Nassar I.N., Rashad M., Hafez M., Norton J.B. (2018). Reclamation of calcareous soil and improvement of squash growth using brewer’s spent grain and compost. J. Saudi Soc. Agric. Sci..

[38] Luo T., Zhu Y., Lu W., Chen L., Min T., Li J., Wei C. (2021). Acidic compost tea enhances phosphorus availability and cotton yield in calcareous soils by decreasing soil pH. Acta Agric. Scand. Sect. B-Soil Plant Sci..

[39] Estrada-Bonilla G.A., Durrer A., Cardoso E.J. (2021). Use of compost and phosphate-solubilizing bacteria affect sugarcane mineral nutrition, phosphorus availability, and the soil bacterial community. Appl. Soil Ecol..

[40] Sánchez Ó.J., Ospina D.A., Montoya S. (2017). Compost supplementation with nutrients and microorganisms in composting process. Waste Manag..

[41] Kiani-Pouya A., Roessner U., Jayasinghe N.S., Lutz A., Rupasinghe T., Bazihizina N., Bohm J., Alharbi S., Hedrich R., Shabala S. (2017). Epidermal bladder cells confer salinity stress tolerance in the halophyte quinoa and Atriplex species. Plant Cell Environ..

[42] Ahmadi S.H., Solgi S., Sepaskhah A.R. (2019). Quinoa: A super or pseudo-super crop? Evidences from evapotranspiration, root growth, crop coefficients, and water productivity in a hot and semi-arid area under three planting densities. Agric. Water Manag..

[43] Rizzello C.G., Tagliazucchi D., Babini E., Rutella G.S., Saa D.L.T., Gianotti A. (2016). Bioactive peptides from vegetable food matrices: Research trends and novel biotechnologies for synthesis and recovery. J. Funct. Foods.

[44] Adel H. (2020). Towards expanding quinoa cultivation in Egypt: The effect of compost and vermicompost on quinoa pests, natural enemies and yield under field conditions. Agric. Sci..

[45] Bazile D., Bertero H.D., Nieto C. (2015). State of the Art Report on Quinoa Around the World in 2013.

[46] Hinojosa L., Gonz’alez J.A., Barrios-Masias F.H., Fuentes F., Murphy K.M. (2018). Quinoa abiotic stress responses: A review. Plants.

[47] Semida W.M., Abdelkhalik A., Rady M.O.A., Marey R.A., Abd El-Mageed T.A. (2020). Exogenously applied proline enhances growth and productivity of drought stressed onion by improving photosynthetic efficiency, water use efficiency and up-regulating osmoprotectants. Sci. Hortic..

[48] Abd El-Mageed T.A., Abdelkhalik A., Abd El-Mageed S.A., Semida W.M. (2021). Co-composted poultry litter biochar enhanced soil quality and eggplant productivity under different irrigation regimes. J. Soil Sci. Plant Nutr..

[49] Shaaban A., Al-Elwany O.A.A.I., Abdou N.M., Hemida K.A., El-Sherif A.M.A., Abdel-Razek M.A., Semida W.M., Mohamed G.F., Abd El-Mageed T.A. (2022). Filter mud enhanced yield and soil properties of water-stressed *Lupinus termis* L. in saline calcareous soil. J. Soil Sci. Plant Nutr..

[50] Hasanuzzaman M., Bhuyan M.H.M.B., Nahar K., Hossain M.S., Al Mahmud J., Hossen M.S., Masud A.A.C., Moumita, Fujita M. (2018). Potassium: A vital regulator of plant responses and tolerance to abiotic stresses. Agronomy.

[51] Malhotra H., Vandana, Sharma S., Pandey R., Hasanuzzaman M., Fujita M., Oku H., Nahar K., Hawrylak-Nowak B. (2018). Phosphorus nutrition: Plant growth in response to deficiency and excess. Plant Nutrients and Abiotic Stress Tolerance.

[52] Meng X., Chen W.W., Wang Y.Y., Huang Z.R., Ye X., Chen L.S., Yang L.T. (2021). Effects of phosphorus deficiency on the absorption of mineral nutrients, photosynthetic system performance and antioxidant metabolism in *Citrus grandis*. PLoS ONE.

[53] Liu J., Shu A., Song W., Shi W., Li M., Zhang W., Li Z., Liu G., Yuan F., Zhang S. (2021). Long-term organic fertilizer substitution increases rice yield by improving soil properties and regulating soil bacteria. Geoderma.

[54] Semida W.M., Abd El-Mageed T.A., Howladar S.M. (2014). A novel organo-mineral fertilizer can alleviate negative effects of salinity stress for eggplant production on reclaimed saline calcareous soil. Acta Hort..

[55] Alghamdi S.A., Al-Ghamdi F.A., El-Zohri M., Al-Ghamdi A.A.M. (2023). Modifying of Calcareous Soil with Some Acidifying Materials and Its Effect on *Helianthus Annuus* (L.) Growth. Saudi J. Biol. Sci..

[56] Kranz C.N., McLaughlin R.A., Johnson A., Miller G., Heitman J.L. (2020). The effects of compost incorporation on soil physical properties in urban soils—A concise Review. J. Environ. Manag..

[57] Wu Q.F., Hu H.B., He L.M. (2020). Effect of *aspergillus niger* strain xf-1 on soil nutrients and growth of *Amorpha fruticosa*. Appl. Ecol. Environ. Res..

[58] Qaswar M., Chai R., Ahmed W., Jing H., Han T., Liu K., Ye X., Xu Y., Anthonio C.K., Zhang H. (2020). Partial substitution of chemical fertilizers with organic amendments increased rice yield by changing phosphorus fractions and improving phosphatase activities in fluvo-aquic soil. J. Soils Sediments.

[59] Abdelkhalik A., Abd El-mageed T.A., Mohamed I.A.A., Semida W.M., Al-elwany O.A.A.I., Ibrahim I.M., Hemida K.A., El-saadony M.T., Abuqamar S.F., El-tarabily K.A. (2023). Soil application of effective microorganisms and nitrogen alleviates salt stress in hot pepper (*Capsicum annum* L.) plants. Front. Plant Sci..

[60] Khan K.S., Ali M.M., Naveed M., Ishaq M., Rehmani A., Waleed M., Ali H.M., Abdelsalam N.R., Ghareeb R.Y., Feng G. (2022). Co-application of organic amendments and inorganic P increase maize growth and soil carbon, phosphorus availability in calcareous soil. Front. Environ. Sci..

[61] Abdelkhalik A., Pascual B., Nájera I., Baixauli C., Pascual-Seva N. (2019). Deficit irrigation as a sustainable practice in improving irrigation water use efficiency in cauliflower under Mediterranean conditions. Agronomy.

[62] Rady M.M., Mossa A.T.H., Youssof A.M., Osman A.S., Ahmed S.M., Mohamed I.A. (2023). Exploring the reinforcing effect of nano-potassium on the antioxidant defense system reflecting the increased yield and quality of salt-stressed squash plants. Sci. Hortic..

[63] Semida W.M., Abd El-mageed T.A., Abdelkhalik A., Hemida K.A., Abdurrahman H.A., Howladar S.M., Leilah A.A.A., Rady M.O.A. (2021). Selenium modulates antioxidant activity, osmoprotectants, and photosynthetic efficiency of onion under saline soil conditions. Agronomy.

[64] Zulfiqar F., Akram N.A., Ashraf M. (2020). Osmoprotection in plants under abiotic stresses: New insights into a classical phenomenon. Planta.

[65] Abd El-mageed T.A., Gyushi M.A.H., Hemida K.A., El-Saadony M.T., Abd El-Mageed S.A., Abdalla H., AbuQamar S.F., El-Tarabily K.A., Abdelkhalik A. (2022). Coapplication of effective microorganisms and nanomagnesium boosts the defenses against salt stress in Ipomoea batatas. Front. Plant Sci..

[66] Martínez-Ispizua E., Calatayud Á., Marsal J.I., Basile F., Cannata C., Abdelkhalik A., Soler S., Valcárcel J.V., Martínez-Cuenca M.-R. (2022). Postharvest changes in the nutritional properties of commercial and traditional lettuce varieties in relation with overall visual quality. Agronomy.

[67] Martínez-Ispizua E., Calatayud Á., Marsal J.I., Cannata C., Basile F., Abdelkhalik A., Soler S., Valcárcel J.V., Martínez-Cuenca M.-R. (2022). The nutritional quality potential of microgreens, baby leaves, and adult lettuce: An underexploited nutraceutical source. Foods.

[68] Zhang Q., Dai W., Dai W. (2019). Plant response to salinity stress. Stress Physiology of Woody Plants.

[69] Baliyan S., Mukherjee R., Priyadarshini A., Vibhuti A., Gupta A., Pandey R.P., Chang C. (2022). Determination of antioxidants by DPPH radical scavenging activity and quantitative phytochemical analysis of *Ficus religiosa*. Molecules.

[70] Sánchez-Navarro V., Zornoza R., Faz Á., Fernández J.A. (2021). Cowpea crop response to mineral and organic fertilization in SE Spain. Processes.

[71] Ye L., Zhao X., Bao E., Li J., Zou Z., Cao K. (2020). Bio-organic fertilizer with reduced rates of chemical fertilization improves soil fertility and enhances tomato yield and quality. Sci. Rep..

[72] Adnan M., Fahad S., Khan I.A., Saeed M., Ihsan M.Z., Saud S., Riaz M., Wang D., Wu C. (2019). Integration of Poultry Manure and Phosphate Solubilizing Bacteria Improved Availability of Ca Bound P in Calcareous Soils. 3 Biotech.

[73] Mohamed A.S., Mohamed M.H.M., Halawa S.S., Saleh S.A. (2023). Partial exchange of mineral N fertilizer for common bean plants by organic N fertilizer in the presence of salicylic acid as foliar application. Gesunde Pflanzen.

[74] Aykul S., Martinez-Hackert E. (2017). Determination of half-maximal inhibitory concentration using biosensor-based protein interaction analysis. Physiol. Behav..

[75] Van Loon W.A., Linssen J.P., Legger A., Voragen A.G. (2006). Anti-radical power gives insight into early lipid oxidation events during frying. J. Sci. Food Agric..

[76] Youssef M.A., Farag M.I.H. (2021). Co-application of organic manure and bio-fertilizer to improve soil fertility and production of quinoa and proceeding Jew’s Mallow crops. J. Soil Sci. Plant Nutr..

[77] Yang Y., Syed S., Mao S., Li Q., Ge F., Lian B., Lu C. (2020). Bioorganic–mineral fertilizer can remediate chemical fertilizer-oversupplied soil: Purslane planting as an example. J. Soil Sci. Plant Nutr..

[78] Chen S., Gao J., Chen H., Zhang Z., Huang J., Lv L., Tan J. (2023). The role of long-term mineral and manure fertilization on P species accumulation and phosphate-solubilizing microorganisms in paddy red soils. Soil.

[79] Adnan M., Fahad S., Zamin M., Shah S., Mian I.A., Danish S., Zafar-Ul-hye M., Battaglia M.L., Naz R.M.M., Saeed B. (2020). Coupling Phosphate-Solubilizing Bacteria with Phosphorus Supplements Improve Maize Phosphorus Acquisition and Growth under Lime Induced Salinity Stress. Plants.

[80] Ponce V.M., Pandey R.P., Ercan S. (2000). Characterization of drought across the climate spectrum. J. Hydrol. Eng. ASCE.

[81] Klute A., Dirksen C. (1986). Hydraulic conductivity and diffusivity: Laboratory methods, in methods of soil analysis: Part 1-physical and mineralogical methods (Soil Science Society of America). Am. Soc. Agron..

[82] Page A.I., Miller R.H., Keeny D.R. (1982). Methods of soil analysis, in Part II. Chemical and Microbiological Methods.

[83] Pikovskaya R. (1948). Mobilization of phosphorus in soil in connection with vital activity of some microbial species. Mikrobiologiya.

[84] Hu X., Chen J., Guo J. (2006). Two phosphate and potassium-solubilizing bacteria isolated from Tianmu Mountain, Zhejiang, China. World J. Microbiol. Biotechnol..

[85] Premono M.E., Moawad A., Vlek P. (1996). Effect of phosphate-solubilizing Pseudomonas putida on the growth of maize and its survival in the rhizosphere. Indones. J. Crop Sci..

[86] Elias F., Woyessa D., Muleta D. (2016). Phosphate solubilization potential of rhizosphere fungi isolated from plants in jimma zone, Southwest Ethiopia. Int. J. Microbiol..

[87] Prajapati K., Modi H.A. (2012). The importance of potassium in plant growth-a review. Indian J. Plant Sci..

[88] Pitt J.I., Hocking A.D. (2009). The ecology of fungal food spoilage. Fungi and Food Spoilage.

[89] White T.J., Bruns T., Lee S., Taylor J. (1990). Amplification and direct sequencing of fungal ribosomal RNA genes for phylogenetics. PCR Protoc. Guide Methods Appl..

[90] Gardes M., Bruns T.D. (1993). ITS Primers with enhanced specificity for basidiomycetes—Application to the identification of mycorrhizae and rusts. Mol. Ecol..

[91] Tamura K., Peterson D., Peterson N., Stecher G., Nei M., Kumar S. (2011). MEGA5: Molecular evolutionary genetics analysis using maximum likelihood, evolutionary distance, and maximum parsimony methods. Mol. Biol. Evol..

[92] Li Y., Zhang S., Lv Y., Zhai H., Cai J., Hu Y. (2022). Linalool, the main volatile constituent from *Zanthoxylum schinifolium* pericarp, prevents growth of *Aspergillus flavus* in post-harvest grains. Food Control.

[93] Idrovo-Novillo J., Gavilanes-Terán I., Angeles Bustamante M., Paredes C. (2018). Composting as a method to recycle renewable plant resources back to the ornamental plant industry: Agronomic and economic assessment of composts. Process Saf. Environ. Prot..

[94] Sosa-Zuniga V., Brito V., Fuentes F., Steinfort U. (2017). Phenological growth stages of quinoa (*Chenopodium quinoa*) based on the BBCH scale. Ann. Appl. Biol..

[95] Meier U., Bleiholder H., Buhr L., Feller C., Hack H., Heß M., Lancashire P.D., Schnock U., Stauß R., Van Den Boom T. (2009). The BBCH system to coding the phenological growth stages of plants–history and publications. J. Kulturpflanzen.

[96] Lichtenthaler H.K., Buschmann C. (2001). Chlorophylls and carotenoids: Measurement and characterisation by UV-VIS. Current Protocols in Food Analytical Chemistry.

[97] Shibaeva T.G., Mamaev A.V., Sherudilo E.G. (2020). Evaluation of a SPAD-502 plus chlorophyll meter to estimate chlorophyll content in leaves with interveinal chlorosis. Russ. J. Plant Physiol..

[98] Lowry O.H., Rosebrough N.J., Farr A.L., Randall R.J. (1951). Protein measurement with the folin phenol reagent. J. Biol. Chem..

[99] Fales F.W. (1951). The assimilation and degradation of carbohydrates by yeast cells. J. Biol. Chem..

[100] Schlegel H.Q. (1956). Die verwertung organischer säuren durch Chlorella im licht. Planta.

[101] Ábrahám E., Hourton-Cabassa C., Erdei L., Szabados L. (2010). Methods for determination of proline in plants. Methods Mol. Biol..

[102] Anderson M.E. (1985). Determination of glutathione and glutathione disulfide in biological samples. Meth. Enzymol..

[103] Jagota S., Dani H. (1982). A new colorimetric technique for the estimation of vitamin C using Folin phenol reagent. Anal. Biochem..

[104] Sauvesty A., Page F., Huot J. (1992). A simple method for extracting plant phenolic compounds. Can. J. For. Res..

[105] Abe N., Murata T., Hirota A. (1998). Novel 1,1-diphenyl-2-picryhy-drazyl-radical scavengers, bisorbicillin and demethyltrichodimerol, from a fungus. Biosci. Biotechnol. Biochem..

[106] AOAC (2012). Official method of analysis. Association of Analytical Chemists.

[107] Jackson M.L. (1967). Soil Chemical Analysis.

[108] Johnson C.M., Ulrich A. (1959). Analytical methods for use in plant analysis. Calif. Agric. Exp. Stat Bull..

[109] AOAC (2005). Official Methods of Analysis of AOAC International.

[110] Singleton V.L., Orthofer R., Lamuela-Raventós R.M. (1999). Analysis of total phenols and other oxidation substrates and antioxidants by means of folin-ciocalteu reagent. Meth. Enzymol..

[111] Liyana-Pathirana C., Shahidi F. (2005). Optimization of extraction of phenolic compounds from wheat using response surface methodology. Food Chem..

[112] Nostro A., Guerrini A., Marino A., Tacchini M., Di Giulio M., Grandini A., Akin M., Cellini L., Bisignano G., Saraçoğlu H.T. (2016). In vitro activity of plant extracts against biofilm-producing food-related bacteria. Int. J. Food Microbiol..

[113] Bartlett M.S. (1937). Properties of sufficiency and statistical tests. Proc. R. Soc. Ser. A.

[114] Casella G. (2008). Statistical Design.

