# Supplementary material for: Compost and Phosphorus/Potassium-Solubilizing Fungus Effectively Boosted Quinoa’s Physio-Biochemical Traits, Nutrient Acquisition, Soil Microbial Community, and Yield and Quality in Normal and Calcareous Soils
Source: Plants (Basel). 2023 Aug 27;12(17):3071. doi: 10.3390/plants12173071 (PMC10489913; doi:10.3390/plants12173071)
Supplement: Supplementary file 1 [file plants-12-03071-s001.zip › plants-2541226-supplementary.pdf]

Table S1. Mean squares in ANOVA (combined ANOVA) for the effect of season, soil type, compost with phosphate (P)-potassium (K)-solubilizing Aspergillus niger (PK+C-AN) level, and their interactions on chlorophylls (i.e., chlorophyll *a*; chl *a*, chlorophyll *b*; chl *b*, total chlorophyll; total chl, and total photosynthetic pigments; TPP), and carotenoids, and biochemical attributes (i.e., total soluble proteins; TSPs, total soluble sugars; TSSs, free proline; FPro, reduced glutathione; rGSH, ascorbic acid; AsA, total phenolics; TPs, and 2,2-diphenyl-1-picrylhydrazyl-scavenging activity; DPPH-SA) in leaves of quinoa in (SI) 2021/22 and (SII) 2022/23 winter seasons.

**S.O.V.**	**DF**	**Chl *a***	**Chl *b***	**Total chl**	**Carotenoids**	**TPP**	**TSPs**	**TSSs**	**FPro**	**rGSH**	**AsA**	**TPs**	**DPPH-SA (%)**
		**(mg cm^{-2})**					**(mg g^{-1} dry weight)**						
Replicate (R)	2	0.013	0.214	0.141	0.002	0.124	0.013	0.457	0.012	0.028	0.094	0.063	0.421
Season (S)	1	0.179^{ns}	0.027^{ns}	0.351^{ns}	0.497^{ns}	0.014^{ns}	0.058^{ns}	0.0214^{ns}	0.058^{ns}	0.056^{ns}	0.056^{ns}	0.213**	0.122^{ns}
R × S (Error)	2	0.808	0.120	0.882	0.041	0.907	0.288	0.019	0.021	0.061	0.084	0.003	0.792
Soil type (ST)	1	39.608**	36.640**	152.44**	16.068**	267.489**	49.601**	74.176**	0.157^{ns}	303.072**	4.930**	655.220**	311.839**
S × ST	1	1.049	3.617	0.771	1.298	0.069	0.381	0.236	0.002	0.003	0.097	0.020	0.090
R × S × ST (Error)	4	1.028	0.883	2.421	0.087	2.739	0.084	0.017	0.064	0.021	0.068	0.050	0.134
PK+C-AN level	5	131.121**	8.008**	198.78**	5.087**	264.801**	135.607**	25.271**	8.102**	94.345**	150.140**	43.055**	2239.624**
S × PK+C-AN	5	4.568	0.626	6.371	0.572	6.235	0.081	0.0465	0.117	0.067	0.069	0.024	1.354
ST × PK+C-AN	5	9.371**	0.792^{ns}	3.372*	1.192**	8.999*	10.276**	4.810**	37.461**	19.749**	20.654**	7.751**	352.407**
S × ST × PK+C-AN	5	1.724	0.368	1.981	0.290	1.521	0.038	0.040	0.034	0.018	0.073	0.018	2.390
R × S × ST × PK+C-AN (Error)	40	2.509	0.360	2.551	0.166	2.81	0.076	0.044	0.043	0.034	0.045	0.039	0.973
Total	71												

* and ** indicate differences at $p \leq 0.05$ and $p \leq 0.01$ probability level, respectively. ns= not significant difference.

Table S2. Mean squares in ANOVA (combined ANOVA) for the effect of season, soil type, compost with phosphate (P)-potassium (K)-solubilizing Aspergillus niger (PK+C-AN) level, and their interactions on leaf macro and micro-nutrients (i.e., nitrogen; N, phosphorus; P, potassium; K^{+}, calcium; Ca^{2+}, iron; Fe^{2+}, zinc; Zn^{2+}, and manganese; Mn^{2+}) contents and seed proximate chemical composition (i.e., crude protein, ash, crude lipid, crude fiber, and Carbohydrates) of quinoa in (SI) 2021/22 and (SII) 2022/23 winter seasons.

		**N**	**P**	**K^{+}**	**Ca^{2+}**	**Fe^{2+}**	**Zn^{2+}**	**Mn^{2+}**	**Crude protein**	**Ash**	**Crude lipid**	**Crude fiber**	**Carbohydrates**
		**($mg\ g^{-1}$ dry weight)**							**(%)**				
Replicate (R)	2	0.001	0.060	0.013	7.514	6.573E-04	0.0006	0.001	0.081	0.025	0.006	0.002	0.307
Season (S)	1	27.572**	0.022^{ns}	35.420**	1.531^{ns}	2.387E+00**	0.0001^{ns}	3.578**	3.373**	0.352**	7.752**	0.553**	58.310**
R × S (Error)	2	0.024	0.046	0.005	1.167	1.232E-03	0.004	0.0004	0.00002	0.00002	0.00002	0.00009	0.0002
Soil type (ST)	1	78.588**	0.300**	263.543**	61.420**	3.293E+01**	2.000**	156.498**	86.559**	1.202**	2.606**	3.345**	417.135**
S × ST	1	3.482	0.00003	0.058	0.031	9.665E+00	0.288	18.352	0.0201	0.0008	0.007	0.002	0.379
R × S × ST (Error)	4	0.608	0.009	0.005	0.069	4.965E-05	0.0006	0.001	0.0181	0.0041	0.022	0.001	0.046
PK+C-AN level	5	18.170**	6.941**	321.478**	258.254**	1.476E+01**	0.5700**	2.311**	2.620**	3.428**	0.093^{ns}	0.329**	9.681**
S × PK+C-AN	5	1.209	0.001	5.920	0.065	7.382E+00	0.080	0.417	0.0006	0.002	0.0003	0.0002	0.006
ST × PK+C-AN	5	2.737**	0.151**	18.477**	1.487^{ns}	4.149E+00**	0.034**	1.041**	2.634**	0.966**	0.213**	0.043**	7.037**
S × ST × PK+C-AN	5	0.701	0.006	31.646	0.115	5.194E+00	0.009	0.172	0.0006	0.0006	0.0006	0.00003	0.006
R × S × ST × PK+C-AN (Error)	40	0.181	0.028	0.014	0.980	9.451E-04	0.002	0.0012	0.009	0.0141	0.049	0.0008	0.131
Total	71												

* and ** indicate differences at $p \leq 0.05$ and $p \leq 0.01$ probability level, respectively. ns= not significant difference.

Table S3. Mean squares in ANOVA (combined ANOVA) for the effect of season, soil type, compost with phosphate (P)-potassium (K)-solubilizing Aspergillus niger (PK+C-AN) level, and their interactions on phytochemicals (i.e., total phenolic compounds; TPC and total flavonoid compounds; TFC), antioxidant activity (i.e., half-maximal inhibitory concentration; IC50 and anti-radical power; ARP), and mineral (i.e., phosphorus; P, potassium; K+, calcium; Ca^{2+}, magnesium; Mg^{2+}, sodium; Na^{2+}, iron; Fe^{2+}, and zinc; Zn^{2+}) contents of quinoa's seeds grown in (SI) 2021/22 and (SII) 2022/23 winter seasons.

**S.O.V.**	**DF**	**TPC**	**TFC**	**IC50 (mg ml^{-1})**	**ARP**	**P**	**K^+**	**Ca^{2+}**	**Mg^{2+}**	**Na^+**	**Fe^{2+}**	**Zn^{2+}**
		**(mg 100^{-1} g dry seed)**				**(g 100^{-1} g dry seed)**						
Replicate (R)	2	0.948	6.264	0.00035	0.0064	0.0030	0.00004	0.0045	3.472E-07	1.389E-06	3.587E-06	2.045E-06
Season (S)	1	71.875**	27.240**	0.0039**	0.1291**	0.0001^{ns}	0.1071**	0.0184^{ns}	1.038E+00**	3.001E-02**	1.413E-01**	1.200E-02**
R × S (Error)	2	0.000	0.004	0.0000001	0.000002	0.00003	0.00001	0.0148	3.472E-07	4.167E-06	6.597E-08	2.545E-06
Soil type (ST)	1	654.546**	294.826**	0.0541**	1.9149**	0.0070**	0.7972**	0.6142**	8.012E-01**	1.242E-01**	1.486E-02**	3.563E-03**
S × ST	1	0.143	0.049	0.000016	0.0006	0.00007	0.00018	0.00004	1.545E-01	2.939E-03	1.427E-02	2.695E-03
R × S × ST (Error)	4	0.546	0.575	0.00005	0.0056	0.0004	0.00002	0.00160	2.431E-06	1.389E-06	5.743E-06	1.280E-05
PK+C-AN level	5	1145.287**	682.250**	0.0433**	1.5076**	0.0837**	0.9725**	0.6705**	3.572E-01**	1.929E-02**	1.143E-01**	3.523E-03**
S × PK+C-AN	5	0.250	0.331	0.00001	0.0004	0.00007	0.0179	0.0009	3.934E-02	3.049E-03	2.541E-02	2.759E-04
ST × PK+C-AN	5	13.888**	17.991**	0.00049**	0.0523**	0.00116**	0.0559**	0.0050^{ns}	1.248E-01**	4.631E-04**	8.789E-03**	2.728E-04**
S × ST × PK+C-AN	5	0.003	0.026	0.0000001	0.00002	0.00002	0.0958	0.0002	2.054E-02	1.412E-03	8.705E-03	4.841E-04
R × S × ST × PK+C-AN (Error)	40	1.312	1.003	0.00012	0.0042	0.0002	0.00004	0.0034	4.722E-06	2.917E-06	8.197E-06	9.978E-06
Total	71											

* and ** indicate differences at $p \leq 0.05$ and $p \leq 0.01$ probability level, respectively. ns= not significant difference.

Table S4. Mean squares in ANOVA (combined ANOVA) for the effect of season, soil type, compost with phosphate (P)-potassium (K)-solubilizing Aspergillus niger (PK+C-AN) level, and their interactions on total microbial community (i.e., phosphorus solubilizing microorganisms; PSMs and *Azotobacter* sp.) at the end of experiment in rhizosphere soil and seed yield and yield-related attributes of quinoa and harvest index (HI) of quinoa in (SI) 2021/22 and (SII) 2022/23 winter seasons.

**S.O.V.**	**DF**	**PSMs**	***Azotobacter* sp.**	**Plant height (cm)**	**Plant dry weight (g)**	**Hectoliter weight (kg hL^{-1})**	**Seed yield**	**Biological yield**	**HI (%)**
		**(cfu× 10^3 g^{-1} soil)**					(t ha^{-1})		
Replicate (R)	2	0.012	0.116	18.514	2.413	1.186	0.077	0.304	21.610
Season (S)	1	0.347**	0.002^{ns}	88.889*	4.180^{ns}	0.239^{ns}	0.463^{ns}	2.673^{ns}	2.210^{ns}
R × S (Error)	2	0.004	0.740	4.264	1.230	0.688	0.065	0.559	9.940
Soil type (ST)	1	18.000**	132.845**	6460.056**	2526.053**	8.103**	4.415**	72.946**	1482.140**
S × ST	1	0.027	0.390	40.500	0.324	51.219	0.073	1.100	270.890
R × S × ST (Error)	4	0.021	0.189	15.861	0.318	0.366	0.015	0.121	26.040
PK+C-AN level	5	30.000**	92.898**	334.256**	233.128**	26.818**	3.090**	12.343**	46.000**
S × PK+C-AN	5	0.029	0.147	1.989	2.941	1.894	0.028	0.257	15.660
ST × PK+C-AN	5	51.600**	28.602**	13.156*	38.116**	2.186**	0.431**	1.477**	28.350^{ns}
S × ST × PK+C-AN	5	0.026	0.482	18.933	2.170	1.720	0.017	0.784	47.370
R × S × ST × PK+C-AN (Error)	40	0.008	0.302	4.858	0.645	0.476	0.022	0.140	13.080
Total	71								

* and ** indicate differences at $p \leq 0.05$ and $p \leq 0.01$ probability level, respectively. ns= not significant difference.

Table S5. Individual effect of soil type and compost with phosphate (P)-potassium (K)-solubilizing *Aspergillus niger* (PK+C-AN) level on leaf chlorophylls, and carotenoids of quinoa grown in (SI) 2021/22 and (SII) 2022/23 winter seasons.

**Treatment**	**Chl *a***	**Chl *b***	**Total chl**	**Carotenoids**	**TPP**
	**(mg cm^{-2})**				
Season (S)					
SI	27.1±0.6a	6.05±0.19a	33.2±0.8a	8.10±0.17a	41.3±0.9a
SII	27.0±0.6a	6.01±0.22a	33.1±0.7a	8.26±0.14a	41.3±0.8a
Soil type (ST)					
Normal (Nor)	27.8±0.6a	6.75±0.18a	34.6±0.7a	8.65±0.11a	43.2±0.8a
Calcareous (Calc)	26.3±0.5b	5.32±0.16b	31.7±0.6b	7.71±0.16b	39.4±0.8b
PK+C-AN level					
PK100%	25.3±0.6d	5.00±0.25d	30.3±0.7e	8.08±0.35c	38.4 ±1.0e
PK100%+C-AN	32.5±0.8a	7.22±0.24a	39.7±0.9a	9.25±0.17a	48.9±1.0a
PK75%+C-AN	28.7±0.5b	6.55±0.28b	35.2±0.6b	8.51±0.08b	43.7±0.6b
PK50%+C-AN	27.3±0.4c	6.20±0.31bc	33.5±0.6c	8.03±0.15c	41.5±0.7c
PK25%+C-AN	26.1±0.4cd	5.94±0.31c	32.0±0.7d	7.93±0.21c	40.0±0.8d
PK0%+C-AN	22.7±0.2e	5.28±0.37d	28.0±0.3f	7.30±0.21d	35.3±0.4f
p-value					
S	0.684^{ns}	0.681^{ns}	0.595^{ns}	0.074^{ns}	0.914^{ns}
ST	0.003**	0.003**	0.001**	<.001**	<.001**
PK+C-AN	<.001**	<.001**	<.001**	<.001**	<.001**

Values are means ± standard error (n=3). ** indicates differences at $p \leq 0.01$ probability level, respectively. ns= not significant difference. Mean values for each factor followed by the same lower-case-letter in each column are not significantly different according to the Duncan test ($p \leq 0.05$). PK100%= 72 kg P_2O_5 ha^{-1} + 60 kg K_2O ha^{-1}, PK75%= 54 kg P_2O_5 ha^{-1} + 45 kg K_2O ha^{-1}, PK50%= 36 kg P_2O_5 ha^{-1} + 30 kg K_2O ha^{-1}, PK25%= 18 kg P_2O_5 ha^{-1} + 15 kg K_2O ha^{-1}, PK0%= 0 kg P_2O_5 ha^{-1} + 0 kg K_2O ha^{-1}, and compost was added with a rate of 20 t ha^{-1}. Chlorophyll a (Chl *a*), chlorophyll b (Chl *b*), total chlorophyll (Total chl), and total photosynthetic pigments (TPP).

Table S6. Individual effect of soil type and compost with phosphate (P)-potassium (K)-solubilizing *Aspergillus niger* (PK+C-AN) level on leaf biochemical attributes of quinoa grown in (S_I) 2021/22 and (S_{II}) 2022/23 winter seasons.

Treatment	TSPs	TSSs	FPro	rGSH	AsA	TPs	DPPH-SA
	(mg g^{-1} dry weight)						(%)
Season (S)							
SI	16.5±0.56a	13.2±0.31a	16.1±0.31a	17.2±0.59a	19.5±0.59a	15.5±0.60a	62.5±2.3a
SII	16.6±0.55a	13.2±0.29a	16.0±0.30a	17.2±0.59a	19.5±0.58a	15.4±0.60a	62.4±2.3a
Soil type (ST)							
Normal (Nor)	15.7±0.53b	12.2±0.14b	16.1±0.29a	19.2±0.62a	19.2±0.75b	18.5±0.40a	60.3±2.6b
Calcareous (Calc)	17.4±0.55a	14.2±0.32a	16.0±0.32a	15.1±0.26b	19.8±0.35a	12.4±0.20b	64.5±1.9a
PK+C-AN level							
PK100%	14.6±0.07d	13.5±0.30c	14.6±0.30c	19.0±0.80b	22.1±0.24b	16.0±0.75b	73.0±1.5b
PK100%+C-AN	21.8±0.07a	15.4±0.62a	16.6±0.60a	22.0±1.33a	25.4±0.59a	18.7±1.28a	81.1±1.0a
PK75%+C-AN	19.1±0.43b	14.0±0.46b	16.4±0.28a	16.5±0.30c	18.6±0.30c	15.9±1.04b	64.9±2.5c
PK50%+C-AN	17.0±0.73c	12.9±0.18d	16.6±0.08a	15.8±0.46d	17.0±0.38e	15.0±1.01c	60.8±2.4d
PK25%+C-AN	14.1±0.15e	12.0±0.18e	16.5±0.34a	15.1±0.41e	17.3±0.34d	13.9±0.73d	49.8±0.7e
PK0%+C-AN	13.0±0.22f	11.3±0.15f	15.6±0.89b	14.7±0.42f	16.5±0.31f	13.4±0.65e	45.0±0.8f
p-value							
S	0.698^{ns}	0.401^{ns}	0.239^{ns}	0.441^{ns}	0.502^{ns}	0.014^{**}	0.502^{ns}
ST	$<.001^{**}$	$<.001^{**}$	0.193^{ns}	$<.001^{**}$	0.001^{**}	$<.001^{**}$	$<.001^{**}$
PK+C-AN	$<.001^{**}$	$<.001^{**}$	$<.001^{**}$	$<.001^{**}$	$<.001^{**}$	$<.001^{**}$	$<.001^{**}$

Values are means ± standard error (n=3). ** indicates difference at $p \leq 0.01$ probability level. ns= not significant difference. Mean values for each factor followed by the same lower-case-letter in each column are not significantly different according to the Duncan test ($p \leq 0.05$). $PK_{100\%}$= 72 kg P_2O_5 ha^{-1} + 60 kg K_2O ha^{-1}, $PK_{75\%}$= 54 kg P_2O_5 ha^{-1} + 45 kg K_2O ha^{-1}, $PK_{50\%}$= 36 kg P_2O_5 ha^{-1} + 30 kg K_2O ha^{-1}, $PK_{25\%}$= 18 kg P_2O_5 ha^{-1} + 15 kg K_2O ha^{-1}, $PK_{0\%}$= 0 kg P_2O_5 ha^{-1} + 0 kg K_2O ha^{-1}, and compost was added with a rate of 20 t ha^{-1}. Total soluble proteins (TSPs), total soluble sugars (TSSs), free proline (FPro), reduced glutathione (GSH), ascorbic acid (AsA), total phenolics (TPs), and 2,2-diphenyl-1-picrylhydrazyl-scavenging activity (DPPH-SA).

Table S7. Individual effect of soil type and compost with phosphate (P)-potassium (K)-solubilizing *Aspergillus niger* (PK+C-AN) level on leaf nutrient (i.e., nitrogen, N; phosphorus, P; potassium, K^+; calcium, Ca^{2+}; iron; Fe^{2+}, zinc; Zn^{2+}, and manganese; Mn^{2+}) contents of quinoa grown in (S_I) 2021/22 and (S_{II}) 2022/23 winter seasons.

Treatment	Macro-nutrient				Micro-nutrient		
	**N**	**P**	**K^+**	**Ca^{2+}**	**Fe^{2+}**	**Zn^{2+}**	**Mn^{2+}**
	**(mg g^{-1} dry weight)**						
Season (S)							
SI	36.6±0.31a	7.61±0.12a	47.1±1.02a	28.0±0.74a	10.5±0.37a	4.70±0.05a	6.36±0.20b
SII	35.3±0.25b	7.64±0.12a	45.7±0.82b	28.3±0.76a	10.2±0.14b	4.69±0.05a	6.80±0.34a
Soil type (ST)							
Normal (Nor)	37.0±0.30a	7.69±0.12a	48.3±0.89a	29.1±0.77a	11.0±0.35a	4.86±0.05a	8.06±0.17a
Calcareous (Calc)	34.9±0.19b	7.56±0.12b	44.5±0.85b	27.2±0.69b	9.67±0.11b	4.53±0.03b	5.11±0.07b
PK+C-AN level							
PK100%	35.1±0.28d	7.23±0.04e	43.0±0.89e	28.4±0.42d	10.2±0.54c	4.47±0.04e	6.38±0.39d
PK100%+C-AN	37.7±0.51a	8.62±0.07a	55.3±1.23a	33.8±0.55a	12.5±0.75a	5.02±0.09a	7.25±0.55a
PK75%+C-AN	37.1±0.53b	8.21±0.06b	49.0±0.73b	31.2±0.33b	10.3±0.19b	4.83±0.07b	7.00±0.58b
PK50%+C-AN	35.9±0.39c	7.75±0.04c	46.5±0.55c	29.8±0.44c	10.1±0.21d	4.74±0.05c	6.42±0.44c
PK25%+C-AN	35.4±0.43d	7.49±0.03d	43.7±1.03d	24.8±0.36e	9.78±0.14e	4.66±0.06d	6.32±0.46e
PK0%+C-AN	34.5±0.35e	6.45±0.08f	40.9±0.41f	20.9±0.24f	9.23±0.12f	4.45±0.05e	6.12±0.46f
p-value							
S	<.001**	0.563ns	<.001**	0.371ns	<.001**	0.876ns	<.001**
ST	<.001**	0.004**	<.001**	<.001**	<.001**	<.001**	<.001**
PK+C-AN	<.001**	<.001**	<.001**	<.001**	<.001**	<.001**	<.001**

Values are means ± standard error (n=3). ** indicates difference at $p \leq 0.01$ probability level. ns= not significant difference. Mean values for each factor followed by the same lower-case-letter in each column are not significantly different according to the Duncan test ($p \leq 0.05$). $PK_{100\%}$= 72 kg P_2O_5 ha^{-1} + 60 kg K_2O ha^{-1}, $PK_{75\%}$= 54 kg P_2O_5 ha^{-1} + 45 kg K_2O ha^{-1}, $PK_{50\%}$= 36 kg P_2O_5 ha^{-1} + 30 kg K_2O ha^{-1}, $PK_{25\%}$= 18 kg P_2O_5 ha^{-1} + 15 kg K_2O ha^{-1}, $PK_{0\%}$= 0 kg P_2O_5 ha^{-1} + 0 kg K_2O ha^{-1}, and compost was added with a rate of 20 t ha^{-1}.

Table S8. Individual effect of soil type and compost with phosphate (P)-potassium (K)-solubilizing *Aspergillus niger* (PK+C-AN) level on proximate chemical composition of quinoa's seeds grown in (S_I) 2021/22 and (S_{II}) 2022/23 winter seasons.

**Treatment**	**Crude protein**	**Ash**	**Crude lipid**	**Crude fiber**	**Carbohydrates**
	**(%)**				
Season (S)					
SI	14.4±0.22a	2.80±0.10a	6.56±0.05a	3.50±0.04b	65.2±0.46b
SII	14.0±0.21b	2.66±0.09b	5.91±0.05b	3.68±0.05a	67.0±0.44a
Soil type (ST)					
Normal (Nor)	15.3±0.09a	2.60±0.10b	6.04±0.07b	3.81±0.04a	63.7±0.24b
Calcareous (Calc)	13.1±0.13b	2.86±0.09a	6.42±0.06a	3.38±0.02b	68.6±0.25a
PK+C-AN level					
PK100%	13.3±0.42d	2.30±0.09e	6.13±0.12a	3.41±0.06f	67.8±1.03a
PK100%+C-AN	14.2±0.56c	3.43±0.08a	6.25±0.12a	3.44±0.06e	65.4±0.97d
PK75%+C-AN	14.3±0.29c	3.13±0.07b	6.29±0.13a	3.53±0.06d	65.8±0.65c
PK50%+C-AN	14.4±0.30b	2.96±0.03c	6.16±0.16a	3.61±0.07c	65.6±0.81cd
PK25%+C-AN	14.6±0.32a	2.51±0.04d	6.37±0.15a	3.74±0.08b	65.8±0.80c
PK0%+C-AN	14.4±0.15b	2.03±0.17f	6.21±0.10a	3.83±0.10a	66.4±0.45b
p-value					
S	<.001**	<.001**	<.001**	<.001**	<.001**
ST	<.001**	<.001**	<.001**	<.001**	<.001**
PK+C-AN	<.001**	<.001**	0.119ns	<.001**	<.001**

Values are means ± standard error (n=3). ** indicates difference at $p \le 0.01$ probability level. ns= not significant difference. Mean values for each factor followed by the same lower-case-letter in each column are not significantly different according to the Duncan test ($p \le 0.05$). $PK_{100\%}$= 72 kg P_2O_5 ha^{-1} + 60 kg K_2O ha^{-1}, $PK_{75\%}$= 54 kg P_2O_5 ha^{-1} + 45 kg K_2O ha^{-1}, $PK_{50\%}$= 36 kg P_2O_5 ha^{-1} + 30 kg K_2O ha^{-1}, $PK_{25\%}$= 18 kg P_2O_5 ha^{-1} + 15 kg K_2O ha^{-1}, $PK_{0\%}$= 0 kg P_2O_5 ha^{-1} + 0 kg K_2O ha^{-1}, and compost was added with a rate of 20 t ha^{-1}.

Table S9. Individual effect of soil type and compost with phosphate (P)-potassium (K)-solubilizing *Aspergillus niger* (PK+C-AN) level on phytochemicals and antioxidant activity of quinoa's seeds grown in (S_I) 2021/22 and (S_{II}) 2022/23 winter seasons.

**Treatment**	**TPC** (mg 100^{-1} g dry seed)	**TFC**	**IC50 (mg ml^{-1})**	**ARP**
Season (S)				
SI	66.6±1.58b	31.4±1.21b	0.42±0.01b	2.42±0.06b
SII	68.6±1.63a	32.7±1.26a	0.44±0.01a	2.50±0.06a
Soil type (ST)				
Normal (Nor)	70.6±1.60a	34.1±1.06a	0.46±0.01a	2.30±0.05b
Calcareous (Calc)	64.6±1.46b	30.0±1.31b	0.40±0.01b	2.63±0.06a
PK+C-AN level				
PK100%	61.6±1.37d	24.9±0.51f	0.39±0.01e	2.18±0.03e
PK100%+C-AN	80.9±1.01a	44.1±0.32a	0.51±0.01a	2.99±0.07a
PK75%+C-AN	71.6±1.17b	37.8±0.79b	0.48±0.01b	2.67±0.08b
PK50%+C-AN	72.3±1.03b	31.9±0.60c	0.45±0.01c	2.58±0.05c
PK25%+C-AN	66.8±0.99c	27.7±0.83d	0.40±0.01d	2.31±0.04d
PK0%+C-AN	52.6±0.40e	26.0±1.23e	0.35±0.01f	2.03±0.04f
p-value				
S	<.001**	<.001**	<.001**	<.001**
ST	<.001**	<.001**	<.001**	<.001**
PK+C-AN	<.001**	<.001**	<.001**	<.001**

Values are means ± standard error (n=3). ** indicate differences at $p \leq 0.01$ probability level, respectively. ns= not significant difference. Mean values for each factor followed by the same lower-case-letter in each column are not significantly different according to the Duncan test ($p \leq 0.05$). $PK_{100\%}$= 72 kg P_2O_5 ha^{-1} + 60 kg K_2O ha^{-1}, $PK_{75\%}$= 54 kg P_2O_5 ha^{-1} + 45 kg K_2O ha^{-1}, $PK_{50\%}$= 36 kg P_2O_5 ha^{-1} + 30 kg K_2O ha^{-1}, $PK_{25\%}$= 18 kg P_2O_5 ha^{-1} + 15 kg K_2O ha^{-1}, $PK_{0\%}$= 0 kg P_2O_5 ha^{-1} + 0 kg K_2O ha^{-1}, and compost was added with a rate of 20 t ha^{-1}. Total phenolic compounds (TPC), total flavonoid compounds (TFC), half-maximal inhibitory concentration (IC_{50}), and anti-radical power (ARP).

Table S10. Individual effect of soil type and compost with phosphate (P)-potassium (K)-solubilizing *Aspergillus niger* (PK+C-AN) level on seed mineral (i.e., phosphorus, P; potassium, K^+; calcium, Ca^{2+}; magnesium; Mg^{2+}, sodium; Na^{2+}, iron; Fe^{2+}, and zinc; Zn^{2+}) contents of quinoa grown in (S_I) 2021/22 and (S_{II}) 2022/23 winter seasons.

Treatment	P	K^+	Ca^{2+}	Mg^{2+}	Na^+	Fe^{2+}	Zn^{2+}
	**(g 100^{-1} g dry seed)**						
Season (S)							
SI	0.37±0.01a	2.59±0.06a	1.52±0.04a	0.96±0.05a	0.29±0.01a	0.90±0.01b	0.45±0.00b
SII	0.37±0.01a	2.51±0.05b	1.55±0.04a	0.72±0.02b	0.25±0.01b	0.99±0.02a	0.48±0.00a
Soil type (ST)							
Normal (Nor)	0.38±0.01a	2.66±0.05a	1.62±0.04a	0.95±0.05a	0.31±0.01a	0.96±0.02a	0.47±0.00a
Calcareous (Calc)	0.36±0.01b	2.45±0.05b	1.44±0.04b	0.74±0.02b	0.23±0.01b	0.93±0.02b	0.46±0.00b
PK+C-AN level							
PK100%	0.35±0.00d	2.36±0.05e	1.53±0.03c	0.76±0.04e	0.25±0.02d	0.87±0.02e	0.45±0.00d
PK100%+C-AN	0.48±0.00a	3.04±0.07a	1.84±0.04a	1.14±0.11a	0.32±0.02a	1.11±0.04a	0.48±0.01a
PK75%+C-AN	0.43±0.01b	2.70±0.04b	1.65±0.03b	0.94±0.07b	0.30±0.01b	1.00±0.02b	0.47±0.01b
PK50%+C-AN	0.41±0.01c	2.55±0.03c	1.60±0.03b	0.81±0.04c	0.27±0.01c	0.95±0.01c	0.46±0.01c
PK25%+C-AN	0.32±0.01e	2.40±0.06d	1.44±0.02d	0.77±0.04d	0.25±0.01d	0.91±0.01d	0.46±0.01c
PK0%+C-AN	0.25±0.01f	2.25±0.02f	1.13±0.03e	0.64±0.01f	0.21±0.02e	0.84±0.01f	0.44±0.00e
p-value							
S	0.189^{ns}	$<.001^{**}$	0.382^{ns}	$<.001^{**}$	$<.001^{**}$	$<.001^{**}$	$<.001^{**}$
ST	0.014^{**}	$<.001^{**}$	$<.001^{**}$	$<.001^{**}$	$<.001^{**}$	$<.001^{**}$	$<.001^{**}$
PK+C-AN	$<.001^{**}$	$<.001^{**}$	$<.001^{**}$	$<.001^{**}$	$<.001^{**}$	$<.001^{**}$	$<.001^{**}$

Values are means ± standard error (n=3). ** indicates difference at $p \leq 0.01$ probability level. ns= not significant difference. Mean values for each factor followed by the same lower-case-letter in each column are not significantly different according to the Duncan test ($p \leq 0.05$). $PK_{100\%}$= 72 kg P_2O_5 ha^{-1} + 60 kg K_2O ha^{-1}, $PK_{75\%}$= 54 kg P_2O_5 ha^{-1} + 45 kg K_2O ha^{-1}, $PK_{50\%}$= 36 kg P_2O_5 ha^{-1} + 30 kg K_2O ha^{-1}, $PK_{25\%}$= 18 kg P_2O_5 ha^{-1} + 15 kg K_2O ha^{-1}, $PK_{0\%}$= 0 kg P_2O_5 ha^{-1} + 0 kg K_2O ha^{-1}, and compost was added with a rate of 20 t ha^{-1}.

Table S11. Individual effect of soil type and compost with phosphate (P)-potassium (K)-solubilizing *Aspergillus niger* (PK+C-AN) level on total microbial community at the end of experiment in rhizosphere soil, seed yield and seed yield-related attributes of quinoa grown in (S_I) 2021/22 and (S_{II}) 2022/23 winter seasons.

**Treatment**	**PSMs**	***Azotobacter* sp.**	**Plant height (cm)**	**Plant dry weight (g)**	**HW (kg hL^{-1})**	**Seed yield**	**Biological [illegible]**	**HI (%)**
	**(CFU× 10^3 g^{-1} soil)**					**(t ha^{-1})**		
Season (S)								
SI	2.43±0.40b	3.73±0.58a	98.3±1.7a	22.7±1.3a	68.7±0.4a	2.41±0.09a	5.17±0.26a	48.2±1.2a
SII	2.57±0.42a	3.72±0.52a	96.1±2.0b	22.2±1.2a	68.8±0.2a	2.25±0.10a	4.78±0.23a	47.8±1.0a
Soil type (ST)								
Normal (Nor)	3.00±0.10a	5.08±0.66a	106.7±0.9a	28.4±0.9a	68.4±0.3b	2.58±0.10a	5.98±0.23a	43.5±0.9b
Calcareous (Calc)	2.00±0.56b	2.37±0.25b	87.8±1.0b	16.5±0.6b	69.0±0.4a	2.09±0.07b	3.97±0.12b	52.5±0.7a
PK+C-AN level								
PK100%	1.50±0.45d	3.30±0.05c	88.6±2.6e	19.8±2.1d	67.6±0.3d	1.91±0.10d	3.98±0.32e	49.0±1.5a
PK100%+C-AN	2.00±0.60c	8.80±1.32a	102.8±3.1a	30.2±2.7a	71.2±0.6a	2.88±0.12a	6.25±0.45a	47.4±1.7ab
PK75%+C-AN	1.50±0.45d	3.80±0.48b	100.8±2.8b	24.5±1.7b	69.4±0.2b	2.81±0.12a	5.76±0.39b	49.7±1.3a
PK50%+C-AN	1.50±0.45d	3.92±0.34b	99.8±2.8b	21.9±1.4c	68.8±0.4c	2.64±0.12b	5.44±0.42c	50.0±2.1a
PK25%+C-AN	3.00±0.03b	1.83±0.37d	97.8±3.0c	20.2±1.2d	68.3±0.3c	2.07±0.08c	4.72±0.26d	44.9±2.5b
PK0%+C-AN	5.50±1.06a	0.70±0.03e	93.5±3.5d	18.0±1.7e	67.0±0.3e	1.70±0.05e	3.70±0.21e	46.9±2.1ab
p-value								
S	0.011**	0.967^{ns}	0.045*	0.207^{ns}	0.616^{ns}	0.117^{ns}	0.160^{ns}	0.684^{ns}
ST	<.001**	<.001**	<.001**	<.001**	0.009**	<.001**	<.001**	0.002**

Values are means ± standard error (n=3). * and ** indicate differences at $p \leq 0.05$ and $p \leq 0.01$ probability level, respectively. ns= not significant difference. Mean values for each factor followed by the same lower-case-letter in each column are not significantly different according to the Duncan test ($p \leq 0.05$). $PK_{100\%}$= 72 kg P_2O_5 ha^{-1} + 60 kg K_2O ha^{-1}, $PK_{75\%}$= 54 kg P_2O_5 ha^{-1} + 45 kg K_2O ha^{-1}, $PK_{50\%}$= 36 kg P_2O_5 ha^{-1} + 30 kg K_2O ha^{-1}, $PK_{25\%}$= 18 kg P_2O_5 ha^{-1} + 15 kg K_2O ha^{-1}, $PK_{0\%}$= 0 kg P_2O_5 ha^{-1} + 0 kg K_2O ha^{-1}, and compost was added with a rate of 20 t ha^{-1}. Phosphorus solubilizing microorganisms (PSMs), hectoliter weight (HW) and harvest index (HI).

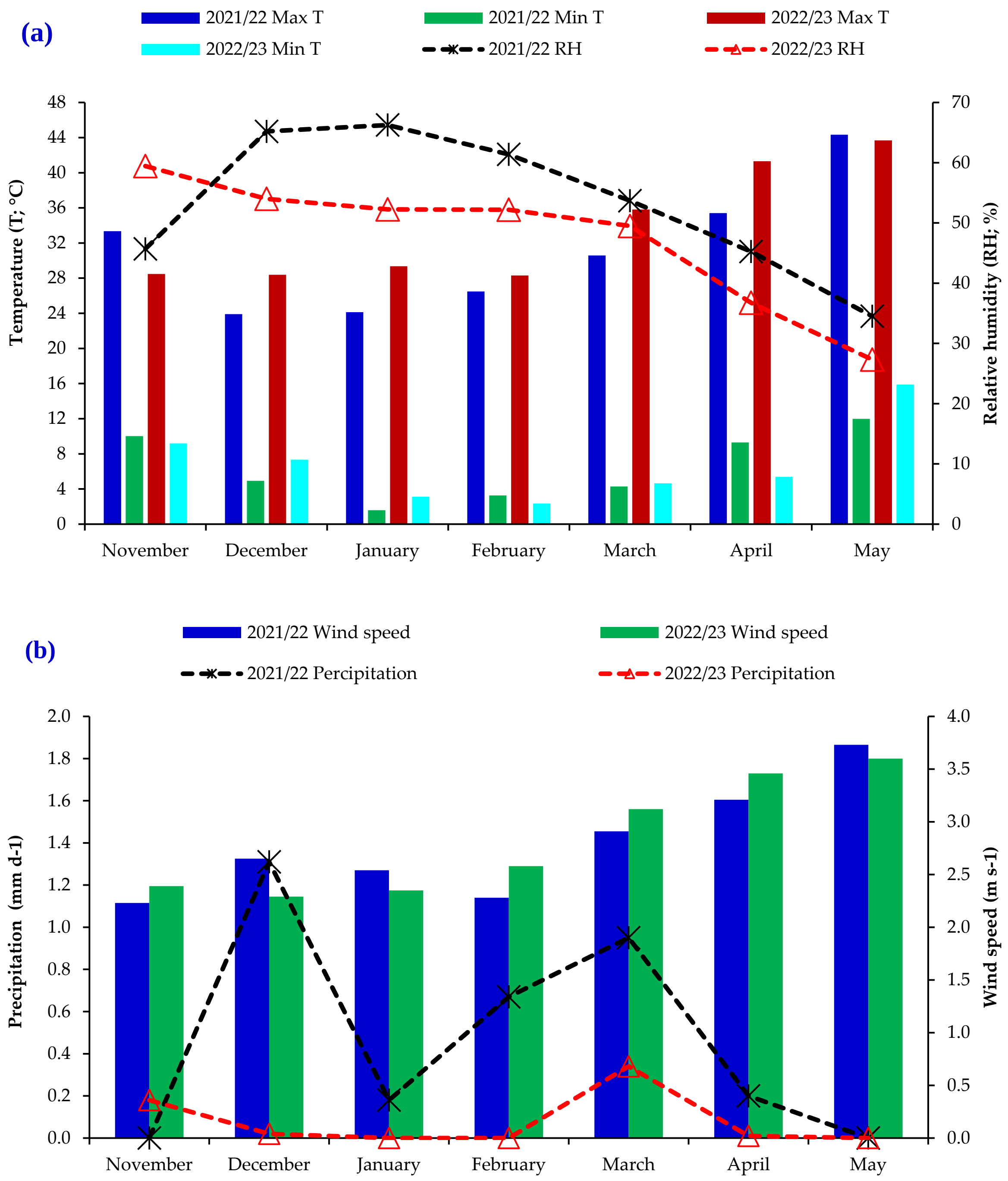

Figure S1. Monthly data of agro-climatic parameters [Maximum (Max) and Minimum (Min) temperatures and relative humidity (a), and precipitation and wind speed (b)] of the experimental location during the 2021/22 and 2022/23 winter growing seasons.
